# Cheyne–Stokes respiration detected via CPAP devices as a digital biomarker for heart failure in obstructive sleep apnoea: systematic review

**DOI:** 10.1093/sleepadvances/zpag042

**Published:** 2026-04-06

**Authors:** Nashe Marshall Mutombe, Kanchana Ekanayake, Chin Moi Chow

**Affiliations:** Faculty of Health and Medicine, University of Sydney, Camperdown, NSW 2006, Australia; Faculty of Health and Medicine, University of Sydney, Camperdown, NSW 2006, Australia; Faculty of Health and Medicine, University of Sydney, Camperdown, NSW 2006, Australia

**Keywords:** Cheyne–Stokes breathing, periodic breathing, ventricular function, left, haemodynamics, sleep apnoea, central, sleep apnoea syndromes, biological markers, stroke volume, telemedicine, prognosis

## Abstract

**Study Objectives:**

To synthesize evidence on the diagnostic, prognostic, surveillance, and monitoring potential of Cheyne–Stokes respiration (CSR) ventilatory patterns, as detected by continuous positive airway pressure (CPAP) devices, in patients with heart failure (HF) or at risk of HF—often with comorbid obstructive sleep apnoea.

**Materials and Methods:**

This systematic review was conducted in accordance with the Preferred Reporting Items for Systematic Reviews and Meta-Analyses (PRISMA 2020) statement. Five electronic databases were searched (1946–2025) for studies examining CSR derived from CPAP airflow signals (CPAP-CSR), and heart-failure outcomes. Data synthesis followed Synthesis Without Meta-analysis guidelines across five domains: presence, burden, morphology, temporal dynamics, and aetiology.

**Results:**

Five studies were included. CPAP-CSR presence was strongly associated with serious cardiac events (adjusted OR 5.74; *p* < .001) at 12 months. CPAP-CSR burden correlated with [B-type natriuretic peptide (BNP), left ventricular ejection fraction (LVEF), and estimated glomerular filtration rate (eGFR)], HF hospitalisations, and serious cardiac events. Morphological features, specifically longer cycle lengths (˃68.9 s), accurately discriminated HF-related CSR (AUC 0.954). Short-term temporal dynamics were highly prognostic; day-to-day instability (SD-CSB%) predicted acute HF decompensation (AUC 0.919), reflecting an inability to maintain normal function. Aetiological stratification revealed that progressive CPAP-CSR burden and serious cardiac events occurred exclusively in the cardiovascular-related CSR subgroups.

**Conclusions:**

CPAP-CSR is consistently associated with heart-failure status and its temporal changes, highlighting its potential as a non-invasive biomarker for disease presence and progression.

## Introduction

### Cheyne–Stokes respiration and heart failure: epidemiology

Heart failure is an escalating global health priority. It is characterized by a dynamic clinical syndrome in which the heart cannot pump enough blood to meet the body’s metabolic requirements, or can do so only at the cost of elevated filling pressures [1]. HF currently affects an estimated 64.3 million people worldwide [2]. Atop the burden of disease, HF poses substantial strain on healthcare systems through high rates of morbidity and hospitalisation, with the lifetime risk of developing the condition for a 45-year-old now reaching approximately 24% [2, 3].

The terms—Cheyne–Stokes breathing (CSB) and Cheyne–Stokes Respiration (CSR)—are often referred to interchangeably, due to historical precedence dating back to the early 19^th^ century when the ventilatory pattern was first described, and in relation to cardiac disease [4, 5]. CSR is a variant of unstable periodic breathing characterized by crescendo-decrescendo ventilatory cycles, punctuated by central apnoeas or hypopnoeas [6, 7]. While CSR is rare in the community—affecting only 0.4% to 1.1% of adults [8], it is highly prevalent in populations with heart failure, where it affects 30%–50% of patients [9]. In heart failure with reduced ejection fraction (HFrEF) registries, approximately 31% of patients with sleep-disordered breathing exhibit central sleep apnoea CSA, while 41% show periodic breathing patterns [10]. Remarkably, prevalence estimates vary significantly by clinical setting i.e. stable chronic HF cohorts exhibit CSR rates around 28% [11] whereas acute inpatient settings report CSR in up to 66% of cases when measured by amount of “time-in-pattern” [12]. This variability underscores the significant influence of patient phenotype (stable vs. decompensated) and the diagnostic metrics applied, such as apnoea-hypopnoea index (AHI)-based thresholds versus periodic-breathing indices [8–12].

### Cheyne–Stokes respiration: aetiology

The apnoeic threshold is the arterial partial pressure of carbon dioxide (PaCO₂) level below which the metabolic drive to breathe is insufficient to maintain ventilation and central apnoea ensues—a key principle which remains true in both pathological central apnoeas and transient central apnoeas observed in healthy individuals [13, 14]. The subsequent apnoea then returns PaCO₂ levels toward stable eupnoeic levels via ongoing systemic metabolism [13–15]. The PaCO₂ reserve represents a stabilising, yet narrow, PaCO₂ buffer [typically 2-6 mmHg during non-rapid eye movement (NREM) sleep] between eupnoeic PaCO₂ and the apnoeic threshold [15, 16].

The ventilatory instability underlying CSR arises from a dysregulated ventilatory control feedback loop, which is governed by the apnoeic threshold, and modulated via peripheral chemoreceptors within the carotid artery and central chemoreceptors within the brainstem [14, 17]. Meanwhile, the impact of changes in ventilation on PaCO₂ is buffered by the body’s CO₂ stores, including gaseous CO₂ in the lung’s functional residual capacity and larger bicarbonate (HCO₃^-^)-based stores in the blood and soft tissues, where the HCO₃^-^/CO₂ system acts as a major physiological buffer [18–20].

In CSR, increased chemoreceptor sensitivity causes breathing to overcompensate for apnoeic increases in PaCO₂ resulting In hyperventilation [16, 21]. This hyper-ventilatory overshoot leads to drops in PaCO₂ below the apnoeic threshold, leading to apnoea, particularly in patients who are predisposed to low PaCO₂ reserve [16, 21], and thus perpetuating the CSR oscillations.

The underlying mechanism of periodic breathing, including CSR, was mathematically formalized by Khoo et al. (1982) using the loop-gain engineering framework which models the interactions between controller gain (chemosensitivity), plant gain (lung and blood-gas dynamics), and circulatory delay [22]. Ultimately, CSR emerges when the overall loop-gain (the product of controller gain and plant gain) exceeds unity i.e. the corrective ventilatory response to a gas-exchange disturbance is larger than the disturbance itself.

### Cheyne–Stokes respiration in heart failure: aetiology

CSR—in both sleep and wakefulness, serves as a potent physiological marker of heart-failure severity reflecting critical failure of ventilatory control stability, and underlying cardiac output [23–25]. In addition to being a symptomatic manifestation, the presence of CSR independently predicts subsequent cardiac death, even in clinically stable patients receiving optimized medical therapy [25, 26].

Patients with HF are particularly predisposed to CSR manifestation, due to inherently increased plant gain, increased controller gain, and delayed circulation [21, 22, 27–29]. First, patients with HF exhibit increased controller gain, where hypersensitivity of their chemoreceptors result in overly heightened ventilatory responses to small rises in PaCO₂ [21, 30]. Second, plant gain may be increased in some patients with heart failure due to reduced PaCO₂ buffering capacity [19, 31]. FRC may be reduced in HF as a result of pulmonary oedema, pleural effusions, and cardiomegaly, thereby reducing lung gas stores [29]. In parallel, lower metabolic rate and carbon dioxide production (V̇CO₂), particularly in patients with HFrEF and low BMI, reduce the body’s CO₂ stores and amplify PaCO₂ fluctuations for a given change in ventilation [19, 31]. These increases in plant gain may promote central apnoea and contribute to CSR in susceptible patients [19, 29, 31, 32].Third, prolonged circulatory delay—an intrinsic hallmark of reduced cardiac output in heart failure—exacerbates CSR by delaying feedback of blood-gas changes from the lungs to the chemoreceptors, thereby increasing overshoot and undershoot in ventilation and influencing cycle length [27, 28]. The convergence of these factors elevates the total loop-gain above unity, manifesting in the self-sustaining hallmark—crescendo-decrescendo pattern of CSR in patients with HF [6, 21, 28].

While CSR is markedly prevalent in patients with HF, it represents a heterogeneous periodic breathing variant with cardiac and non-cardiac aetiologies [29, 33, 34]. In non-cardiac, or high loop-gain CSR, the oscillations typically manifest with shorter, more variable CSR cycle lengths and increased pattern irregularity [33, 34]. In contrast, heart-failure-related CSR is characterized by longer, smoother, and highly regular cycles that closely track circulatory delay and underlying haemodynamic impairment [29, 33, 34]. Mixed or intermediate breathing patterns frequently occur in atrial fibrillation and in HF with preserved ejection fraction, wherein concomitant obstructive and central mechanisms often interact to shape respiratory instability [35, 36].

### Cheyne–Stokes respiration in sleep: aetiology

CSR is most prevalent during non-rapid eye movement (NREM) sleep compared with rapid eye movement (REM) sleep and wakefulness [23]. During NREM sleep, withdrawal of the wakefulness and neuro-modulatory respiratory drive renders ventilation almost entirely dependent on chemical control (PaCO₂ levels), with a small PaCO_2_ reserve between eupnoea and the apnoeic threshold. Under these conditions, relatively small perturbations in PaCO₂ are sufficient to precipitate central apnoeas [15, 16]. In contrast, CSR typically attenuates or disappears during REM sleep, where irregular, state-dependent non-chemical respiratory drive stabilizes ventilation and overrides the unstable feedback loop governing chemical control [27].

Notably, transient arousals amplify ventilatory instability by provoking acute hyperpnoea and PaCO₂ washout, thereby increasing the likelihood of post-arousal central apnoea and perpetuating CSR oscillations [7]. Subsequent integrative models incorporated these sleep-state-dependent mechanisms within the loop-gain framework, demonstrating that heightened chemosensitivity, low arousal threshold, and prolonged circulatory delay interact to sustain CSR during sleep [14, 37, 38]. Importantly, CSR in HF is observed in NREM sleep where it is amplified by the sleep state, and persistence of CSR across all sleep stages or into wakefulness reflects profoundly destabilized ventilatory control and is associated with worse clinical prognosis, serving as a marker of advanced HF [21, 23].

### Emergent CSR during CPAP therapy in patients with heart failure and comorbid obstructive sleep apnoea: mechanisms of therapy benefit, clinical outcomes, residual AHI and emergence of central apnoeas

In patients with HF and obstructive sleep apnoea (OSA) (a prevalent comorbidity), the primary physiological action of CPAP is upper-airway splinting, resulting in suppression of obstructive respiratory events [39, 40]. By reducing intermittent hypoxaemia, arousal-related sympathetic activation, and exaggerated negative intrathoracic pressure swings, CPAP can lessen ventricular afterload and preload stress and, in selected cohorts, improve haemodynamic indices and cardiac function [39, 41]. Additional benefits on diastolic function and natriuretic peptide profiles have been reported, particularly in HF with preserved or mildly reduced ejection fraction, although findings remain heterogeneous and appear dependent on underlying ventilatory control stability and disease phenotype [42, 43]. In patients with HF and central ventilatory instability, relief of upper-airway obstruction may unmask or accentuate a central breathing pattern, such that residual AHI during CPAP predominantly reflects CSR or central sleep apnoea [44, 45].

### CPAP devices in the detection of CSR: validation

A 2009 multi-centre validation study demonstrated that the ApneaLink algorithm, applied in the ResMed AirSense 10 devices, accurately identified CSR (sensitivity 87.1%, specificity 94.9%) [46]. The second of the two CPAP devices in this systematic review, Philips DreamStation and its predecessor, the System One, apply an on-board firmware algorithm named Digital Auto-Trak. Digital Auto-Trak detects “Periodic Breathing” by identifying waxing-waning flow cycles (40-90s) [47], and it does so in accordance with the AASM waveform criterion for scoring CSR [48]. In a validation study for residual AHI, Gagnadoux et al. (2017) assert that “It has not only the ability to detect airway obstruction from respiratory effort related arousals (RERAs) to hypopneas and apneas, but also to identify CSR”, [47] although specific sensitivity metrics for CSR are not independently reported, as with the ResMed algorithm validation.

### Contemporary heart-failure monitoring and surveillance: efficacy

Contemporary HF management has increasingly shifted toward pre-symptomatic detection of rising haemodynamic stress and congestion, with the objective of enabling earlier therapeutic escalation before overt clinical decompensation or hospitalisation occurs, a strategy supported by guideline recommendations and reinforced by evidence from haemodynamic monitoring trials and remote surveillance studies [49, 50]. A central proof-of-concept for this strategy is haemodynamic surveillance using implantable pulmonary artery pressure sensors [50]. The CHAMPION trial demonstrated that pulmonary artery pressure—guided management with the CardioMEMS system significantly reduced HF hospitalisations and enabled earlier, targeted intensification of diuretic and vasodilator therapy in ambulatory patients with chronic HF [50]. Subsequent studies, including GUIDE-HF and MONITOR-HF, further established the clinical relevance of continuous haemodynamics monitoring across broader populations of patients with HF [51–53].

Observational studies using CPAP telemonitoring data show that CSR burden frequently increases with worsening congestion and may regress following effective decongestion, optimisation of HF pharmacotherapy, or correction of structural cardiac disease, thus supporting CSR interpretation as a dynamic haemodynamic biomarker rather than a fixed trait [54–56]. Building on this concept, telemonitoring cohorts have demonstrated that higher proportions of sleep time spent in CSR (CSR%) or periodic breathing (PB%), longer CSR cycle length, and within-patient increases in these metrics were associated with incident HF, HF exacerbation, and serious cardiac events, indicating that CPAP device-derived CSR metrics (CPAP-CSR) may function as a passive, longitudinal early-warning signal of cardiovascular instability [56, 57] The limitations with the highly sensitive—implantable pulmonary artery pressure sensors (e.g. CardioMEMS) include that of an invasive procedure, device- and service-infrastructure requirements, and a clinical workflow that typically concentrates use in selected, higher-risk patients [25, 50, 51, 53].

In contrast, CPAP-derived CSR monitoring is non-invasive and intrinsically scalable because it leverages an already-deployed home therapy platform to generate high-frequency, longitudinal respiratory signals without additional patient-facing procedures, creating a pragmatic route for opportunistic monitoring and surveillance in CPAP-treated populations [46, 56, 57]. The principal limitation in CPAP-CSR monitoring is that ventilatory instability is an indirect biomarker i.e. CPAP-CSR can reflect haemodynamic stress and circulatory delay in HF, however it does not directly measure those parameters, or contemporary diagnostic parameters in HF [56–59]. Furthermore, while CSR characteristics in HF are well described, CSR has multiple phenotypes including high loop-gain OSA, and non-cardiac phenotypes which can represent false signals if not sufficiently filtered out from heart-failure-related—CPAP-CSR [56–59].

Altogether, CPAP-CSR metrics potentially represent a continuous, non-invasive, remarkably low-burden, low-cost, and high-coverage, early-warning signal that can prioritize clinical review. Moreover, these metrics can trigger confirmatory evaluation and enrich decision-making when interpreted alongside symptoms, weight, natriuretic peptides, and other surveillance streams. Thus, they serve timely identification and early intervention in at-risk patients in HF management.

### Evidence gaps

Despite converging physiological rationale and encouraging cohort-level study evidence, there remains no rigorous, device and algorithm-agnostic synthesis quantifying the diagnostic or prognostic utility of CPAP-CSR for HF care. Observational data from CPAP cohorts indicate that CSR presence and burden—derived from nightly flow signals—are associated with HF status and, in some cases, adverse cardiac outcomes. Critically, key CPAP-CSR metrics known to relate to HF pathophysiology, such as CSR percentage, cycle length (a surrogate of circulatory delay), pattern regularity, and temporal variability have never been systematically evaluated for their ability to discriminate HF presence, severity, or clinical trajectory. No study has formally compared the performance of these metrics across device platforms or algorithmic approaches, nor assessed their potential diagnostic yield against current HF risk-stratification tools. Consequently, contemporary HF and sleep-disordered breathing guidelines do not incorporate CPAP-CSR indices as actionable markers for screening, risk stratification, or dynamic monitoring of pre-existing HF, incident HF, or HF exacerbation despite the widespread use of CPAP among patients with HF. This gap underscores the need for broader investigation and methodological consolidation. Currently lacking is a consolidated and methodologically consistent evaluation that compares CPAP-CSR metrics across studies, assesses their diagnostic and prognostic value beyond standard CPAP therapy, and establishes clinically interpretable CSR thresholds suitable for prospective testing in diverse HF-OSA populations.

### Objectives

The primary objective of this systematic review is to evaluate the diagnostic and prognostic accuracy of CPAP flow-signal—detected CSR, in identifying HF and continuously monitored HF-related clinical outcomes across real-world clinical populations (including new-onset, worsening, or acute exacerbations). Focus will be placed on whether CPAP-CSR improves detection of HF presence, refines risk stratification for adverse HF events, and provides incremental predictive value in alignment with standard clinical, imaging, or biomarker-based approaches.

The secondary objective is to determine which specific CPAP-CSR metrics, such as cycle length and CSR percentage, most effectively distinguish heart-failure-related patterns from non-heart-failure-related patterns. The review will also examine temporal dynamics, including short-term variability and burden instability over time, to evaluate their utility in signalling imminent HF events.

## Materials and Methods

### Protocol and registration

This systematic review was conducted in accordance with the Preferred Reporting Items for Systematic Reviews and Meta-Analyses (PRISMA 2020) statement [60] and the Synthesis Without Meta-analysis reporting guideline [61]. Authors submitted the study protocol for PROSPERO database registration prior to data extraction (registration ID—CRD420251171330). Certainty of evidence was appraised using the Grading of Recommendations Assessment, Development and Evaluation (GRADE) framework [62].

### Information sources and search strategy

Comprehensive searches were conducted in MEDLINE (OvidSP), Embase (OvidSP), CINAHL (EBSCOhost), Scopus (Elsevier), and Web of Science Core Collection (Clarivate) for studies that were published between 1 January 1946 to 2 June 2025. No start date restriction was applied, as preliminary scoping identified no prior systematic review addressing this specific research question. Developed in consultation with an academic librarian (KE), the search strategy was structured around three core concepts i.e. (1) sleep-disordered breathing and obstructive sleep apnoea; (2) Cheyne–Stokes respiration and CPAP-based physiological monitoring; and (3) heart failure and cardiovascular outcomes. For each concept, we identified a comprehensive list of search terms, including (medical subject headings) terms and free-text keywords, to ensure all relevant literature was captured. Citation searching was also employed to identify missed or non-indexed material. Searches were limited to English-language publications.

### Eligibility criteria

#### Inclusion criteria

Population: Adults (≥18 years) diagnosed with OSA or related sleep-disordered breathing and established on CPAP therapy.

Exposure: Presence or burden of Cheyne–Stokes respiration, periodic breathing, or central sleep apnoea identified exclusively via proprietary CPAP algorithms or the analysis of raw CPAP airflow signals.

Outcomes: Quantitative associations between CPAP-CSR metrics and HF-related endpoints, including prevalent HF, incident HF, acute or chronic exacerbations, HF-hospitalisations, serious cardiac events, or established physiological markers of cardiac function (e.g., BNP, LVEF, or eGFR).

#### Exclusion criteria

Technical Modalities: Studies where CSR was derived solely from polysomnography (PSG) or alternative positive airway pressure modalities, such as adaptive servo-ventilation (ASV).

Study Design: Publications that did not provide original analytical evidence, including literature reviews, systematic reviews, meta-analyses, editorials, viewpoints, and commentaries.

Descriptive Reports: Non-analytical study designs, such as case reports and case series, which lack the statistical power to evaluate prognostic performance.

Language and Format: Non-English language publications and conference abstracts with insufficient methodological detail.

#### Selection process

**Figure 1:**
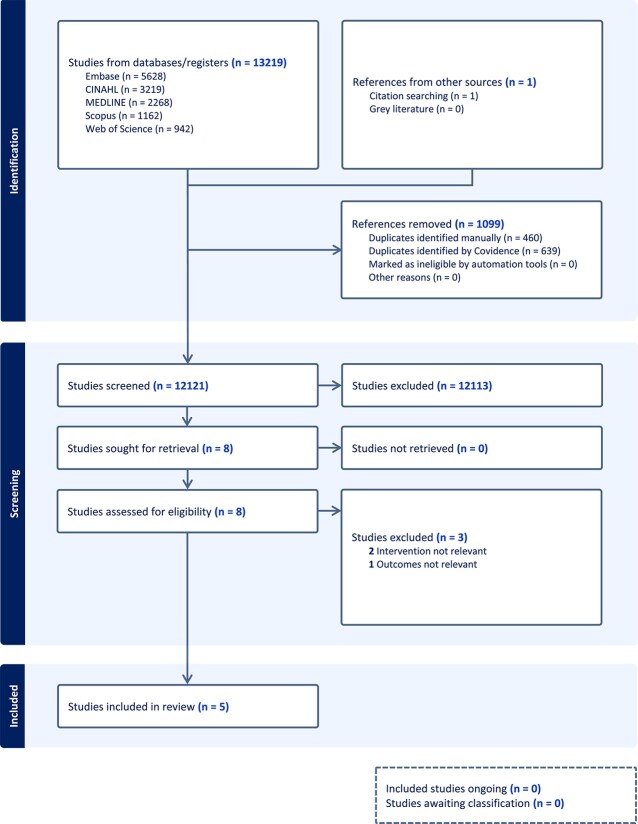
Flowchart of literature identification and selection process (PRISMA flow diagram).

Two reviewers independently screened all titles, abstracts and full texts. Discrepancies were resolved by consensus. The selection process, including reasons for full-text exclusions, is summarized in the PRISMA flow diagram (Figure 1).

#### Data extraction process

Data were extracted independently by two reviewers using a structured template that captured study design, participant characteristics, CPAP device model, algorithm used for PB detection, CSR metric type, comparator groups, follow-up duration, statistical methods and all reported effect estimates. Extracted outcomes included the relationship between CPAP-CSR metrics and HF endpoints or physiological indices of cardiac function. Discrepancies in extraction were resolved by consensus.

#### Risk of bias assessment

Risk of bias was assessed using the Joanna Briggs Institute (JBI) checklists for observational studies [63]. Both reviewers assessed each study independently and reached consensus on all ratings.

#### Reporting and documentation

The search strategy, data extraction sheets, JBI risk-of-bias assessments and PRISMA (2020) checklist are available in the supplementary materials i.e. Text File S1, Dataset S1, and Tables S1 and S2, respectively.

#### Operational definition of Cheyne–Stokes respiration in CPAP-derived data

According to the AASM, Cheyne–Stokes breathing is a pattern comprising at least three consecutive central apnoeas or hypopnoeas separated by a crescendo-decrescendo ventilatory pattern, typified by cycle lengths between 45 and 90 seconds, and the presence of at least five central events per hour, sustained for a minimum of two hours [64, 65].

In all included studies, CSR was identified exclusively via proprietary CPAP algorithms applied to the CPAP airflow signals. Peer-reviewed validation studies, including Gagnadoux et al. (2017) in the Journal of Clinical Sleep Medicine, and Weinreich et al. (2009), assert the ability of the Philips-Respironics and ResMed algorithms (respectively) in identifying CSR. Table 1 summarizes each algorithm’s evaluation against PSG.

**Table 1:** Validation: proprietary algorithms versus PSG scoring for CPAP devices in included studies [48, 56, 57, 66, 67]

Feature Studies	ResMed algorithm Midelet et al. (2023); Prigent et al. (2022); Prigent et al. (2025)	Philips respironics algorithm Saito & Takamatsu, (2022); Ullah et al. (2023)
Algorithm	**(Proprietary CSR Classifier)**	**Digital Auto-Trak**
Method	Analyses flow shape for specific CSR pattern post-recording.	Real-time monitoring of “waxing/waning” cycles (40-90s)—as in AASM criterion.
Validation paper	*(Weinreich* et al.*, 2009)*	*(Gagnadoux* et al.*, 2017)*
Reported metric	**Sensitivity: 87.1% / Specificity: 94.9%**	Strong correlation (ICC >0.90) for residual events; no specific % reported for CSR.
		
Output name	“Cheyne–Stokes Respiration”	“Periodic Breathing”

Boldface in Table 1 identifies the proprietary algorithm/classifier names, the key validation performance data most relevant to this review, and the included studies matched to each of the algorithm/classifier names. The studies constitute the column headings.

Abbreviations: AASM: American Academy of Sleep Medicine; CSR: Cheyne–Stokes Respiration; ICC: Intraclass Correlation Coefficient.

Importantly, both algorithms classify this waveform in accordance with the AASM waveform criterion for scoring CSR [48]. For these reasons, CSR, CSB and PB derived from these CPAP devices were assessed as expressions of the same waveform construct—here termed CPAP-derived Cheyne–Stokes respiration (CPAP-CSR).

#### Rationale for not performing meta-analysis and data preparation and transformation

Meta-analysis was deemed inappropriate for several reasons. First, CPAP-CSR metrics varied substantially across studies in their definitions, temporal windows, and computational derivations, prohibiting meaningful numerical pooling. Second, outcome measures differed widely, ranging from serious cardiac events (SCEs) to HF hospitalisation, exacerbation, incident HF, and surrogate markers—B-type natriuretic peptide, left ventricular ejection fraction, and estimated glomerular filtration rate—with inconsistent follow-up durations. Third, study designs varied (prospective telemonitoring, retrospective cohorts, analytic waveform studies), and statistical models were not comparable. Finally, proprietary CPAP algorithms differed across manufacturers, introducing non-quantifiable measurement heterogeneity. These factors altogether violated assumptions required for meta-analytic synthesis.

Instead, for each metric type, we extracted the following: effect size (if reported), direction of effect, statistical significance, and any accompanying variability measures (IQR, SD, confidence intervals). Where only descriptive comparisons were presented, we used authors’ reported analyses to determine effect direction. No transformations or missing-data imputation was performed.

#### Grouping for synthesis

Studies were grouped according to CPAP-CSR metric type, defined a priori to reflect the physiologically distinct ways in which PB manifests in CPAP-CSR. These metric categories encompassed measures which captured (1) whether CPAP-CSR was detected; (2) burden, which quantified either the proportion of sleep time spent in CSR or the number of CSR-positive nights; (3) morphology, which described structural characteristics of the waveform such as cycle length and inter-cycle variability; (4) temporal instability, which reflected day-to-day variability or longer-term change in CPAP-CSR over time; and (5) aetiology, which distinguished CSR attributed to cardiovascular causes from CSR arising from non-cardiac mechanisms. Grouping studies according to these metric families allowed the synthesis to align closely with physiologically meaningful dimensions of CSR behaviour while enabling consistent comparison across heterogeneous designs and outcome measures.

### Criteria for deciding which studies contributed to each synthesis group

Each study contributed to one or more synthesis groups based on the CPAP-CSR metrics reported. For example, studies reporting cycle length or multifeature waveform analyses contributed to morphological synthesis, whereas studies reporting CSR+ nights or PB% contributed to burden synthesis. Studies reporting temporal variation (e.g. SD-CSB%, year-to-year change) contributed to temporal-instability synthesis. The allocation of studies to groups was determined independently by two reviewers, with discrepancies resolved by consensus.

### Approach to summarising direction of effect

Metrics were interpreted as indicating an increased risk or association with HF in accordance with their underlying physiological meaning, recognising that directionality is case-dependent. In some instances, a lower value reflects reduced CSR burden and therefore a lower risk of HF outcomes, whereas in other cases, particularly for certain morphological features—a lower value may represent a pattern more characteristic of HF-related CSR. The directions of effect for each metrics are presented in the: Summary of Effects by Reported Outcomes. Situations in which, estimates crossed the null, or where estimates were accompanied by wide confidence intervals, or yielded *p*-values ≥0.05, were regarded as demonstrating no clear association. Where studies reported both unadjusted and adjusted analyses, adjusted estimates were prioritized for interpretation. In studies reporting multiple CSR metrics simultaneously e.g. those incorporating both morphological and burden-based measures, each metric was classified independently and allocated to the synthesis grouping most relevant to its conceptual domain.

## Results

### Study selection

The search strategy retrieved 13 220 records across five databases. After removing 1099 duplicates, 12 121 records underwent title and abstract screening. Eight full-text articles were assessed, of which three were excluded because CSR was not derived from CPAP signals or because cardiac outcomes were not reported. Five studies met all eligibility criteria and were included in the qualitative synthesis: Ullah et al. (2023), Prigent et al. (2022), Prigent et al. (2025), Midelet et al. (2023), and Saito and Takamatsu (2022). The complete selection process is presented in the PRISMA flow diagram (Figure 1).

### Study characteristics

The five included studies were all observational and enrolled adults with OSA or other sleep-disordered breathing who were established on CPAP therapy at the time CSR was assessed. Sample sizes ranged from 23 participants in the waveform-analysis study [66] to 555 participants in the AlertApnée telemonitoring [56]. The AlertApnée studies [48, 56] and the Midelet et al. (2023) [66] study used ResMed devices with automated CSR detection, while the Saito and Takamatsu (2022) [57] and Ullah et al. (2023) [67] studies used Philips Respironics devices—also with automated CSR detection. Follow-up ranged from 180 days [67] to two years [48]. Across the dataset, outcomes comprised serious cardiac events, acute and chronic heart-failure exacerbations, HF-related hospitalisation, all-cause mortality and HF severity markers (BNP, LVEF, eGFR), as well as discrimination between HF-related and non-HF periodic breathing. Study characteristics are summarized in Tables 2 and 3.

**Table 2:** Summary of included studies—part (a) [48, 56, 57, 66, 67]

Study	Design & setting	Participants	Device & AASM—CSR criteria followed
Prigent et al. (2022) (Alertapnée Y1)	Prospective cohort: CPAP telemonitoring-triggered diagnostic protocol.	n = 555 adults with OSA on CPAP; CSR^+^ n = 74; CSR^-^ n = 481.	ResMed AirSense 10; (AASM criterion (a) only).
Prigent et al. (2025) (Alertapnée Y2)	Prospective telemonitoring follow-up of Y1 CSR+ cohort; aetiology-stratified analysis.	n = 66 CSR+ from Y1; stratified into CVD-CSR, obstructive-event CSR, leak-related CSR, medication-related CSR, unknown.	ResMed AirSense 10; (AASM criterion (a) only).
Midelet et al. (2023)	Prospective observational study; Cross-sectional CPAP-CSR waveform analysis + ML discrimination of HF-related CSR vs non-HF CSR.	N = 23 patients; 139 CSR episodes (HF = 78; non-HF = 61).	ResMed AirSense 10; (AASM criterion (a) only).
Saito & Takamatsu. (2022)	Retrospective case–control; CPAP-equivalent PB features from PAP downloads and raw waveforms.	n = 33 HF-event patients (11 AHF; 22 CHF exacerbations) + 149 matched at-risk controls (all ≥1% CSB% baseline; 16.8% with stable HF).	Philips Respironics DreamStation; (AASM criterion (a) only).
Ullah et al. (2023)	Retrospective cross-sectional review of medical records; PB% and PAP adherence extracted from CPAP downloads.	n = 115 Veterans with OSA + HF (HFrEF n = 41; HFnmEF n = 74).	Philips Respironics DreamStation; (AASM criterion (a) only).

Abbreviations: CSR^+^: Patients who exhibited Cheyne–Stokes respiration; CSR^-^:Patients who did not exhibit Cheyne–Stokes respiration; AHF: Acute Heart Failure; AUC: Area Under the Curve; BNP: B-type Natriuretic Peptide; CI: Confidence Interval; CL: Cycle Length; CSR: Cheyne–Stokes Respiration; CVD: Cardiovascular Disease; eGFR: estimated Glomerular Filtration Rate; HF: Heart Failure; AHF: Acute Heart Failure; CHF: Chronic Heart Failure; HFrEF: HF with reduced Ejection Fraction; HFnmEF: Heart Failure with non-reduced Ejection Fraction; LR: Logistic Regression; LVEF: Left Ventricular Ejection Fraction; ML: Machine Learning; OR: Odds Ratio; PB: Periodic Breathing; SCE: Serious Cardiac Event; SD-CSB%: Standard Deviation of Daily Cheyne Stokes Breathing Percentage.

**Table 3:** Summary of included studies—part (b) [48, 56, 57, 66, 67]

Study	Exposure (CSR metric)	Comparator	Outcomes measured	Key findings
Prigent et al. (2022) (Alertapnée Y1)	*Incident CPAP-CSR+ presence* detected via telemonitoring after AHI alert.	Patients without detected CPAP-CSR.	**Primary:** SCEs: acute HF, arrhythmia requiring medical intervention.	Incident CSR strongly associated with SCE: Unadjusted OR 13.66 (95% CI 5.18-38.9; *p* < .01). Adjusted OR 5.74 (95% CI 2.08-16.83; *p* < .001) at 12 months. CSR+ patients older with more CV disease. Detection bias noted.
Prigent et al. (2025) (Alertapnée Y2)	Y2 incident CPAP-CSR+ presence; CSR+ nights; CSR%; aetiology classification.	Y2 patients without detected CPAP-CSR; CVD-CSR vs non-CVD CSR aetiology strata.	**Primary:** SCEs during second year. **Secondary:** Change in CSR burden.	All seven Y2 SCEs occurred exclusively in CVD-CSR stratum. CSR+ nights significantly increased Y1 → Y2 only in the 21 CVD-CSR (19 → 37; *p* = .006). CSR+ nights predicted Y2 SCE regardless of CSR%/night (OR 1.05 per night; 95% CI 1.01-1.09; *p* = .017). Higher CPAP-CSR burden in the Y2 SCE+ group i.e. median CSR+ nights (48/90 IQR-35 vs 9.5/90 IQR-27.8, *p* = .012) and the median of mean CSR% (CSR%) (13.8% IQR13.7 vs 6.1% IQR-4.5, *p* = .008).
Midelet et al. (2023)	Morphological CSR features: cycle length; breath duration; inter-cycle variability; amplitude modulation; big breath amplitude ratio; EIAR; CSR episode duration.	HF-CSR vs non-HF CSR (obstruction, leak-related, renal, medication-related).	**Primary:** Discrimination of HF-CSR via logistic regression. **Secondary:** ML performance.	Significant features: ↑ cycle length OR 1.14 (1.07-1.21); ↓ breath duration OR 0.57 (0.35-0.91); ↓ inter-cycle variability OR 0.41 (0.20-0.84). HF prediction ML: accuracy 0.72; F1 0.72; recall 0.78. Big breath ratio not statistically significant but remains clinically relevant. EIAR not statistically significant.
Saito & Takamatsu (2022)	Temporal variability: SD-CSB% across 30 days (10 × 3-day means); Cycle length (CL).	HF events (AHF, CHF exacerbation) vs stable ≥1% CSB% controls.	**Primary:** HF event prediction (ROC). **Secondary:** CSB trajectory.	SD-CSB% higher pre-event (AHF 6.3 ± 3.8; CHF 8.2 ± 4.6) vs controls 1.8 ± 2.1 (*p* < .001). CL longer in HF: stable CHF 79.3 s; exacerbation 85.1 s; controls 59.5 s (*p* < .001). CL extends significantly in CHF exacerbation—(79.3 s → 85.1 s; *p* = .004). Near term HF prediction AUCs: SD-CSB% (0.919 sensitivity 87.9%; specificity 88.6%) (Cut off - 3.275); CL - (0.954 86.4% sensitivity and 90.9% specificity) Cut off (68.9 seconds). (10 -30-day alert signal for AHF and CHF).
Ullah et al. (2023)	PB% (30-day average).	HFrEF vs HFnmEF; high vs low PB%.	**Primary:** HF hospitalisation (180 days). **Secondary:** mortality; PAP adherence.	HFrEF had 2.6-fold higher PB% (24.16% vs 8.64%; *p* < .001). PB% correlated with BNP, LVEF, eGFR and hospitalisation. PB% predicted HF admission. PAP adherence associated with less hospitalisation and mortality.

Boldface in Table 3 identifies the column headings, and the primary and secondary outcome labels for the key HF-related findings/effect estimates most relevant to the synthesis

Abbreviations: CSR^+^: Patients who exhibited Chyeyne-Stokes respiration; CSR^-^:Patients who did not exhibit Cheyne–Stokes respiration; AHF: Acute Heart Failure; AUC: Area Under the Curve; BNP: B-type Natriuretic Peptide; CI: Confidence Interval; CL: Cycle Length; CSR: Cheyne–Stokes Respiration; CVD: Cardiovascular Disease; eGFR: estimated Glomerular Filtration Rate; HF: Heart Failure; CHF: Chronic Heart Failure; HFrEF: HF with reduced Ejection Fraction; HFnmEF: Heart Failure with non-reduced Ejection Fraction; LR: Logistic Regression; LVEF: Left Ventricular Ejection Fraction; ML: Machine Learning; OR: Odds Ratio; PB: Periodic Breathing; SCE: Serious Cardiac Event; SD-CSB%: Standard Deviation of Daily Cheyne Stokes Breathing Percentage.

### Population representativeness

Across all studies, the review population is best characterized as adults on long-term CPAP therapy with a high cardiometabolic risk profile, reflecting the typical population for whom CPAP is prescribed and in whom HF risk is elevated. The samples collectively captured a broad spectrum of HF phenotypes, including HFrEF, HFmrEF, and HFpEF, as well as both acute and chronic HF decompensation. Participants were predominantly older and male, with women substantially under-represented across all studies. Geographically, the studies comprised single-centre cohorts from Japan (Saito and Takamatsu), France (the AlertApnée cohorts and the Midelet et al., study), and the United States (Ullah et al.,). By design, all participants were already receiving CPAP therapy, meaning that HF patients with untreated sleep-disordered breathing were not represented. While this limits generalisability to the broader HF population, the included samples reflect those CPAP users at greatest risk of HF development or deterioration i.e. older adults with OSA and variable cardiovascular comorbidities.

### Risk of bias: JBI

**Figure 2:**
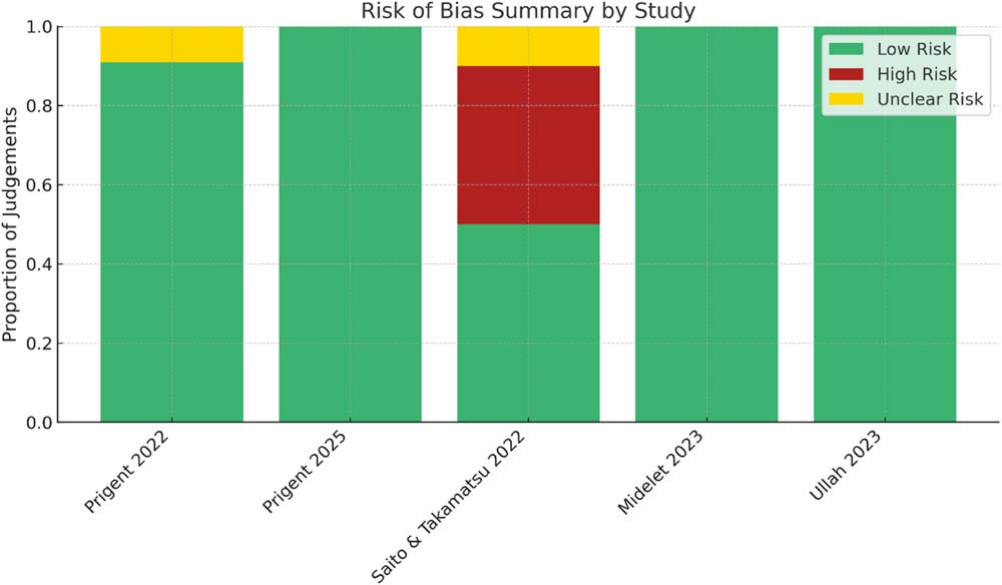
Summary risk of bias assessments for all included studies.

Risk of bias across studies ranged from low to moderate-high. The risk of bias assessments for all included studies is summarized in (Figure 2).

**Figure 3:**
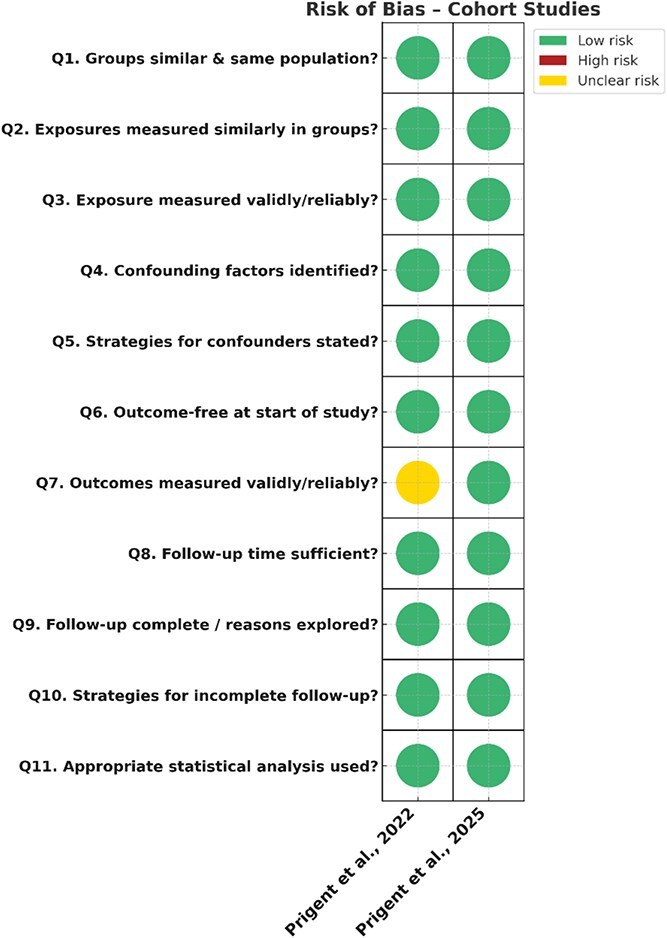
Risk of bias assessments for the cohort studies.

The two AlertApnée studies (Prigent et al., 2022; 2025) were rated as low risk due to clear eligibility criteria, comprehensive outcome definitions, structured telemonitoring protocols and appropriate adjustment for key confounders. In Prigent et al. (2022) however, the assessment of SCEs differed fundamentally, between patients who received a CPAP-triggered alert and those who did not, thus introducing an inherent and potentially significant detection bias. Alert-positive patients underwent a standardized diagnostic testing, including clinical review, electrocardiography, N-terminal pro-B-type natriuretic peptide testing, and echocardiography, whereas alert-negative patients were assessed retrospectively from medical records. Significantly disproportionate intensity of assessment increases the likelihood that SCEs were more frequently identified in the alert group because clinicians were prompted to look for them. As a result, the higher event rate observed among alert-positive patients may partly reflect differential surveillance rather than true differences in underlying cardiac risk. The risk of bias assessments for these cohort studies are summarized in (Figure 3).

**Figure 4:**
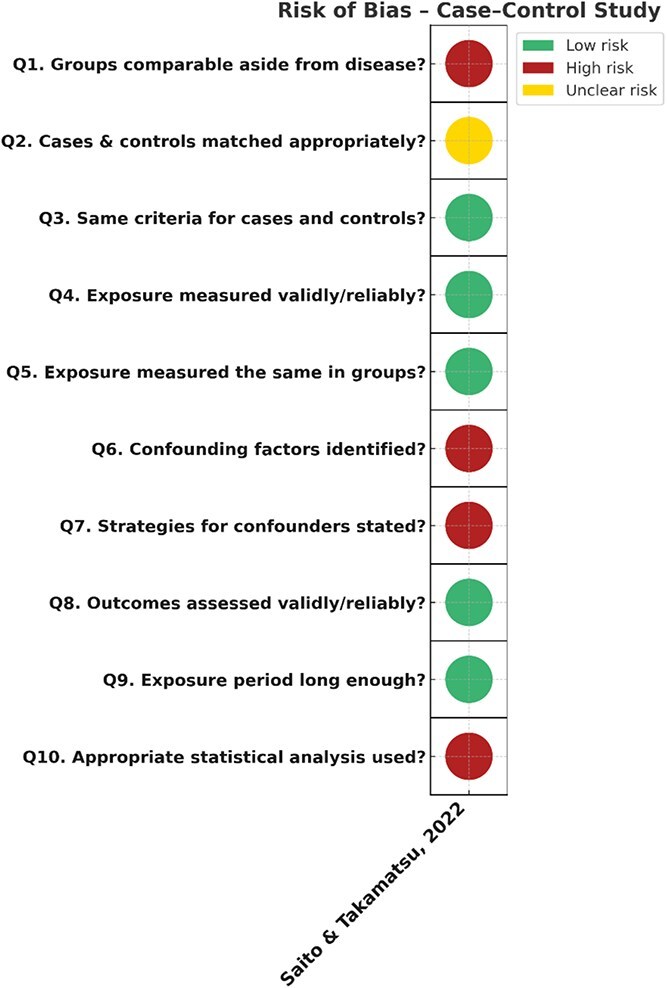
Risk of bias assessment for the case–control study.

The case–control analysis by Saito and Takamatsu (2022) was judged to be at moderate to high risk. The HF and non-HF groups differed meaningfully in baseline characteristics, such as age, comorbidities, and OSA severity, and these confounders were not fully addressed. Furthermore, matching was only done partially and applied to a subset of comparisons. No multivariable regression was performed to adjust for these imbalances, which is a notable limitation for a case–control design. In addition, the statistical methods used to compare temporal changes in SD-CSB% were insufficiently reported, thus making it unclear to decipher how group-level differences were handled analytically. Selection bias is also present, as the control group was defined by CSB% ≥ 1%, a criterion tied directly to the exposure of interest. However, it is arguable that the CSB% ≥ 1% criterion stratified for (at-risk) comparability, but this is not reported. Finally, cycle length was derived from a single night of waveform data. While its consistency with short-term variability measures provides internal coherence, cycle length varies significantly on a nightly basis such that the study’s sampling cannot be relied upon to reflect short/long term trends. Taken together, these issues mean that the reported relationships should be viewed as suggestive rather than definitive. The risk of bias assessment for this case–control study is summarized in (Figure 4).

**Figure 5:**
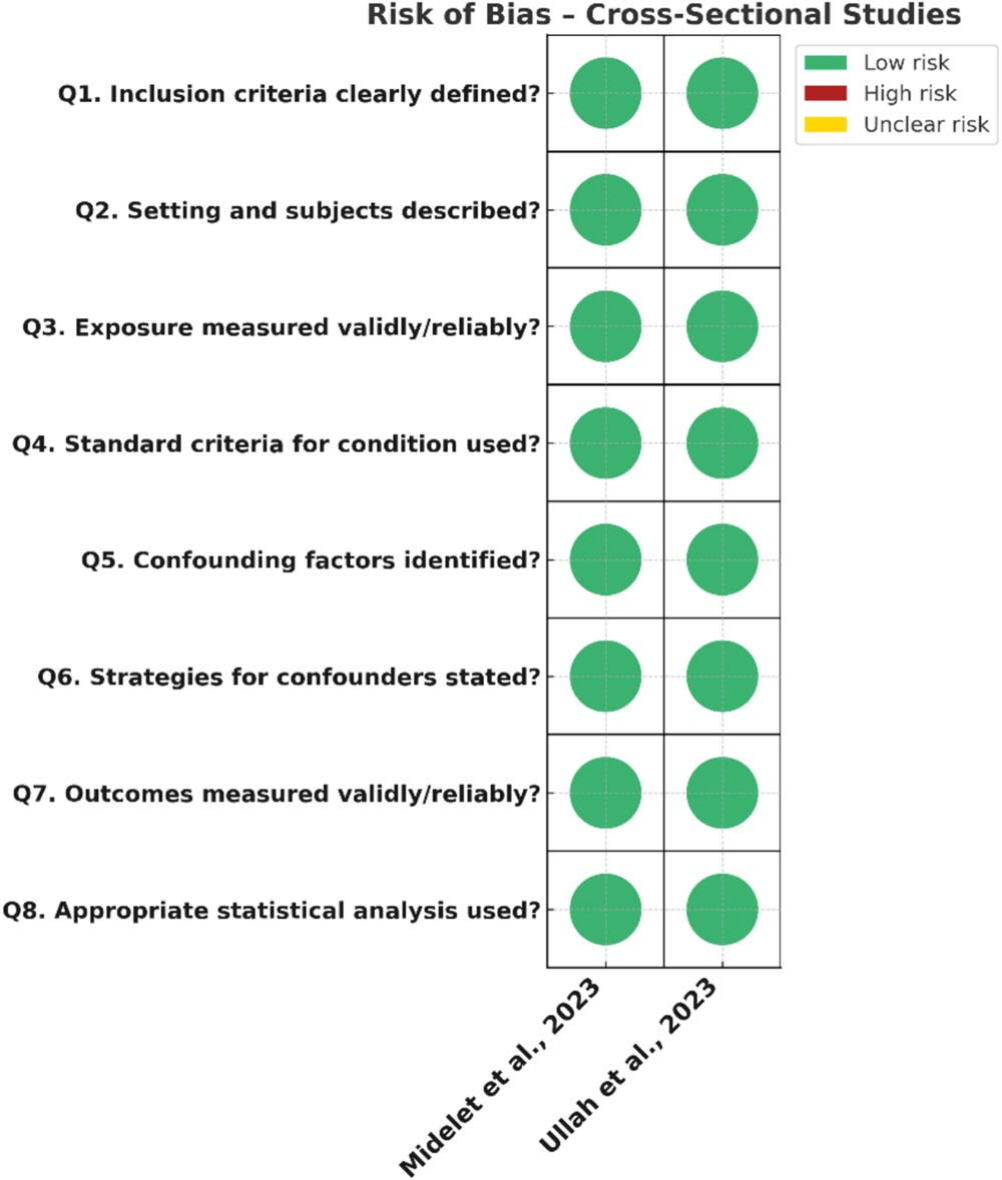
Risk of bias assessments for the cross-sectional studies.

Ullah et al. (2023) and Midelet et al. (2023) were judged to be at moderate risk of bias, largely because of limited adjustment for confounding variables and modest sample sizes. Furthermore, Ullah et al. (2023) employed retrospective data collection. The risk of bias assessments for these cross-sectional studies are summarized in (Figure 5).

### Study results by CPAP-CSR metric domains

#### Study contributions to CPAP-CSR metric syntheses

Each study contributed to one or more CPAP-CSR metric domains. No study contributed to all five domains simultaneously, but together they provided complementary coverage of presence, burden, morphology, temporal dynamics and aetiology-specific CSR. Prigent et al. (2022) contributed to the presence domain, examining incident CSR detection as a binary exposure and its association with serious cardiac events. Prigent et al. (2025) contributed to the burden, temporal and aetiology domains by reporting CSR-positive nights and CSR% over 90-day and two-year intervals, and by stratifying CSR episodes as cardiovascular or non-cardiovascular in origin. Ullah et al. (2023) contributed to the burden domain through PB% as a continuous CSR burden metric linked to HF severity markers, HF hospitalisation and mortality. Midelet et al. (2023) contributed to the morphology domain via multi-feature waveform analysis distinguishing HF-related CSR from non-HF periodic breathing. Saito and Takamatsu (2022) contributed to the burden, morphology and temporal domains by reporting CSB%, cycle length and day-to-day variability in CSB% before acute and chronic heart-failure events. Outcomes included HF status (presesence/absence), SCEs, acute and chronic HF exacerbations, HF-related hospitalisation and surrogate markers of HF severity. Tables 4 and 5 map and define this study’s operational CPAP-CSR Metric Domains respectively.

**Table 4:** Mapping of included studies to CPAP-CSR metric domains, and outcomes [48, 56, 57, 66, 67]

Study	Presence	Burden	Morphology	Temporal dynamics	Aetiology	Primary outcomes
Prigent 2022 (AlertApnée)	✔	-	-	-	-	Serious cardiac events (SCEs)
Prigent 2025 (AlertApnée Y2)	-	✔	-	✔ (long-term)	✔	SCEs; longitudinal CSR progression—(cross-sectioned)
Ullah 2023	-	✔ (PB%)	-	-	-	HF hospitalisation; mortality; HF severity markers
Midelet 2023	-	-	✔	-	-	HF vs non-HF discrimination
Saito & Takamatsu 2022	-	✔ (CSB%)	✔ (CL)	✔ (instability)	-	Acute HF, chronic HF exacerbation vs controls

Abbreviations: CPAP: continuous positive airway pressure; CSR: Cheyne–Stokes respiration; CSB: Cheyne–Stokes breathing; PB: periodic breathing; CL: cycle length; HF: heart failure; SCEs: serious cardiac events.

**Table 5:** CPAP-CSR metric domains, and definitions [48, 56, 57, 66, 67]

Metric category	Operational definition	Studies using this metric
Presence (Binary CPAP-CSR detection)	CPAP algorithm detects **CSR/CSB/PB** at any point during monitoring (incident or persistent).	Prigent 2022
Burden metrics	**CSR-positive nights** across defined monitoring windows (30-90 days); **CSR%/PB%/CSB%** as proportion of nightly sleep spent in CPAP-CSR.	Prigent 2025; Ullah 2023; Saito 2022
Morphology metrics	Waveform descriptors including **cycle lengthbreath durationinter-cycle variabilitymodulation amplitudebig breath amplitude ratio, EIAR; CSR episode duration**.	Midelet 2023; Saito & Takamatsu 2022
Temporal dynamics	Short-term: **SD-CSB%** across consecutive windows; day-to-day variability. Long-term: change in **CSR-positive nights** across months-years.	Saito 2022; Prigent 2025
Aetiology classification	CSR episodes categorized as **CVD-CSR** vs **non-CVD CSR** (mask leak, obstruction, medication-related, unknown).	Prigent 2025

Boldface in Table 5 identifies the column headings, and the specific CPAP-CSR variables that operationalise each metric domain as they were directly synthesised across studies.

Abbreviations: CPAP: continuous positive airway pressure; CSR: Cheyne–Stokes respiration; CSB: Cheyne–Stokes breathing; PB: periodic breathing; CVD: cardiovascular disease; EIAR: expiratory-inspiratory amplitude ratio; SD: standard deviation.

#### CPAP-CSR presence and burden as markers of heart-failure risk

Across studies, CPAP-CSR presence and burden were consistently associated with HF severity and adverse outcomes. In the prospective telemonitoring cohort by Prigent et al. (2022), incident CPAP-CSR identified at the time of an AHI-triggered alert was strongly predictive of SCEs. 20.3% of patients who manifested CSR (CPAP-CSR+) experienced a SCE compared with only 2.0% of CSR negative patients (CPAP-CSR-), with univariable odds ratio (at 12 months) of 13.66 (95% CI 5.18-38.9; *p* < .01). After adjusting for age, dyslipidaemia and cardiovascular comorbidities, CPAP-CSR remained an independent predictor of SCEs (adjusted OR 5.74; 95% CI 2.08-16.83; *p* < .001). Baseline cardiovascular disease was more prevalent in the CPAP-CSR+ group, yet the persistence of the association even in adjusted models indicates that incident CPAP-CSR conveys prognostic information beyond pre-existing risk profiles.

Burden-based measures demonstrated a similar pattern. In the two-year follow-up of the AlertApnée cohort (Prigent et al., 2025), the number of CSR+ nights over a 90-day window was substantially higher in those who later experienced SCEs (48 vs 9.5; *p* = .012), as was the nightly CSR% (13.8% vs 6.1%; *p* = .008). Each additional CSR-positive night increased the odds of SCEs by 5% (OR 1.05; 95% CI 1.01-1.09; *p* = .017). Similar associations were found in the retrospective cohort by Ullah et al. (2023), wherein PB% derived from nightly CPAP data was significantly higher in patients with reduced ejection fraction (24.16% vs 8.64%; *p* < .001) and correlated with BNP (r = 0.447; *p* < .01), LVEF (r = −0.423; *p* < .01) and eGFR (r = −0.246; *p* < .01). PB% predicted HF-related hospitalisation during 180-day follow-up, while PAP adherence was independently protective against both hospitalisation (IRR per 10% increase = 0.78; *p* < .001) and mortality (OR 0.86; *p* = .043).

Across all studies, CPAP-CSR presence and burden behaved as robust indicators of HF severity and near-term cardiac risk.

### Morphological features of CPAP-CSR distinguishing HF from non-HF patterns

Morphological characteristics of CPAP-CSR cycles provided a more specific layer of discrimination between HF-related and non-HF-related—CPAP-CSR. In the CPAP-CSR waveform analysis by Midelet et al. (2023), HF-related CPAP-CSR was defined by longer cycle lengths, shorter breaths, and reduced inter-cycle variability. In multivariable logistic regression, cycle length remained strongly associated with HF-related—CPAP-CSR (OR 1.14 per 1-s increase; 95% CI 1.07-1.21), whereas average breath duration (OR 0.57 per 1-s increase; 95% CI 0.35-0.91), and inter-cycle variability (OR 0.41 per 1-unit increase; 95% CI 0.20-0.84).

Importantly, modulation amplitude showed OR 0.01; 95% CI 0.00-0.24 suggesting that higher values of the variable are associated with lower odds of HF aetiology. However, Midelet et al. (2023) contrarily described “greater variation” in modulation amplitude as characteristic of CSR in HF. This discrepancy may be due to the authors’ decision to prioritize the “medical meaning” of the features and the expert labelling performed by two sleep physicians (Section 2.2.2) over a technically significant but physiologically paradoxical multivariable result. Because the multivariable model yielded an extreme inverse relationship that contradicted both the authors’ qualitative observations and the univariable data (*p* = .85), they appear to have dismissed the result as a likely artefact of limited statistical power (n = 23) rather than a true independent association. In consideration of the discrepancy, and the authors explicitly asserting that—variation of the inspiration amplitude was not associated with any CSR trigger in their logistic regression model—despite the results published in the study’s figure-number-6, this review disqualifies the modulation amplitude result (OR 0.01) as disputed. This interpretation represents a methodological inference rather than an explicit conclusion of the study.

Morphological findings aligned with Saito and Takamatsu (2022), who reported significantly longer CSB cycle lengths in chronic HF than in pair-matched at-risk controls with CSB% > 1% (all *p* < .001), with further prolongation during CHF exacerbation (79.3 s vs 85.1 s; *p* = .004). A threshold of 68.9 seconds differentiated HF-related CSR from controls with high accuracy (AUC 0.954). Together, these studies demonstrate that CPAP-CSR morphological features are not only descriptive but also contain discriminative information that distinguishes HF-driven CPAP-CSR from non-cardiac phenotypes.

### Temporal dynamics of CPAP-CSR in relation to heart-failure decompensation

Increasing temporal variability/ temporal increases in standard deviation (SD) of CPAP-CSR% over short periods, and progression over longer intervals, was linked to clinical deterioration. In Saito and Takamatsu’s study, day-to-day instability in CSR burden, expressed as SD-CSB%, increased sharply in the lead-up to HF events. SD-CSB% differed significantly between HF cases and high-risk controls (*p* < .001), rising abruptly within the 10 days before acute HF presentations, and more gradually over 30 days in chronic exacerbations. The study’s AUROC analysis deducted that a SD-CSB% threshold of 3.275 identified near-term decompensation, with a high discriminatory accuracy (AUC 0.919). Cycle length also lengthened as patients transitioned from stable to exacerbation states (79.3 to 85.1 seconds; *p* = .004), and both values were significantly longer than those of matched controls (*p* < .001).

Longer-term trends—as captured in the year-two AlertApnée follow-up study—showed that all the patients who went on to experience SCE in Y2, had also exhibited markedly increased CSR+ nights in Y2, rising from 19 to 37 nights (*p* = .006). Across both short and long-term observations, spanning from days to years, increases in CPAP-CSR burden over time, were consistently associated with heart-failure deterioration.

### Aetiology-stratified CPAP-CSR (CVD versus non-CVD)

The patients who exhibited significant increases in CSR+ nights in the AlertApnée Y2 follow-up were exclusively—those whose CSR was determined to be of cardiovascular aetiology (CVD-CPAP-CSR). The median CSR-positive nights, and median CSR%, in the final 90-day window of Y2, in the CVD-CPAP-CSR—compared to the non-CVD-CPAP-CSR group showed: median CSR+ nights (48/90 IQR-35 vs 9.5/90 IQR-27.8, *p* = .012), and median of mean CSR% (13.8% IQR13.7 vs 6.1% IQR-4.5, *p* = .008). The CVD-CPAP-CSR group exhibited significantly higher CPAP-CSR burden in Y2. Importantly, all SCEs occurred exclusively in the same CVD-CPAP-CSR group, while no events were observed in patients whose CSR originated from non-cardiac causes such as mask leak, respiratory artefact, medication-related ventilatory instability or residual obstruction. These burden measures were derived from the Y2 comparison between SCE and non-SCE groups.

These findings indicate that the prognostic value of CPAP-CSR is optimized upon delineating the underlying aetiology of the CPAP-CSR, distinguishing cardiovascular-related CSR (CVD-CSR) from other CPAP-CSR subgroups of non-CVD-CSR aetiology. Heart-failure-related CPAP-CSR exhibits trends of progressive burden accumulation and temporal instability that aligned with haemodynamic deterioration, whereas CPAP-CSR of non-cardiac aetiology do not follow similar trajectories and do not demonstrate associations with cardiac risk. Aetiological stratification, therefore, enhances the specificity of CPAP-CSR metrics and reduces the noise introduced by CPAP-CSR which may be regarded as artifact. Summaries of effects by outcomes, and by CPAP-CSR metric domains are outlined in Tables 6 and 7, respectively.

**Table 6:** Summary of effects by reported outcomes [48, 56, 57, 66, 67]

Outcome domain	Study evidence	Direction & strength of association	Notes
Serious cardiac events (SCEs)	Prigent 2022; Prigent 2025	↑ Presence: OR 13.66 → 5.74 adjusted (at 12 months). ↑ Burden: CSR nights & CSR% markedly higher in SCE+. Temporal: long-term burden observed only in CVD-CSR.	Very strong and consistent.
HF exacerbation / acute HF	Saito 2022 (High risk of bias)	SD-CSB% sharply ↑ pre-event; CL ↑; CSR burden ↑ vs controls.	High short-term predictive value.
HF hospitalisation	Ullah 2023	↑ PB% predicts 180-day HF hospitalisation; correlates with BNP, LVEF.	Moderate-strong association.
HF severity markers (BNP, LVEF, eGFR)	Ullah 2023	↑ PB% correlates strongly with all markers.	Burden reflects severity gradient.
HF vs Non-HF discrimination	Midelet 2023; Saito 2022	Morphology discriminates HF-CSR from non-HF; (OR): (a) ↑cycle length - 1.14 (95% CI [1.07, 1.21]), (b) ↓ average breath duration - 0.57 (95% CI [0.35, 0.91]), and (c) ↓ inter-cycle variability 0.41 (95% CI [0.2, 0.84]).	High discriminative validity.

Abbreviations: BNP: B-type Natriuretic Peptide; CI: Confidence Interval; CL: Cycle Length; CSB: Cheyne–Stokes Breathing; CSR: Cheyne–Stokes Respiration; eGFR: estimated Glomerular Filtration Rate; HF: Heart Failure; LVEF: Left Ventricular Ejection Fraction; OR: Odds Ratio; PB: Periodic Breathing; SCEs: Serious Cardiac Events; SD-CSB%: Standard Deviation of Daily Cheyne Stokes Breathing Percentage.

**Table 7:** Summary of effect by CPAP-CSR metric domains (SWiM synthesis Table) [48, 56, 57, 66, 67]

Metric type	Magnitude of association with heart-failure-related outcomes	Consistency across studies	Interpretive summary
Presence	Strong positive association with SCEs (OR 13.66 unadjusted; 5.74 adjusted).	High (1/1 study).	Incident CPAP-CSR represents abrupt destabilisation of respiratory-cardiac coupling and signals imminent cardiac risk.
Burden	Consistently higher in HF groups; predicts SCEs, HF hospitalisation and reduced LVEF; dose–response relationship (OR 1.05 per CSR night).	High (3/3 studies).	Greater CSR/PB/CSB burden reflects more severe HF physiology and increased downstream cardiac risk.
Morphology	HF groups show longer cycles OR 1.14, shorter breaths OR 0.57, lower inter-cycle variability OR 0.41. Cycle length predicts HF with (AUC 0.954).	High (2/2 studies).	HF-related CSR is structurally distinct and recognisable in CPAP data; morphology increases diagnostic specificity.
Short-term temporal instability	Abrupt rise in SD-CSB% prior to acute HF and chronic exacerbation (*p* < .001; AUC 0.919).	High (1/1 study).	Day-to-day instability anticipates decompensation and may provide an early-warning signal.
Long-term trajectory	CSR-positive nights increase over years only in CVD-CSR; strongly predictive of SCEs (*p* = .006).	High in CVD-CSR (1/1).	Progressive CSR accumulation reflects chronic HF deterioration rather than non-HF ventilatory artefacts.
Aetiology-stratified behaviour	All SCEs occur in CVD-CSR subgroup; no adverse events in non-CVD CSR.	Universal	CSR carries prognostic meaning only when driven by cardiovascular pathophysiology.

Abbreviations: AUC: Area Under the Curve; CI: Confidence Interval; CPAP: Continuous Positive Airway Pressure; CSB: Cheyne–Stokes Breathing; CSR: Cheyne–Stokes Respiration; CVD: Cardiovascular Disease; HF: Heart Failure; LVEF: Left Ventricular Ejection Fraction; OR: Odds Ratio; PB: Periodic Breathing; SCEs: Serious Cardiac Events; SD-CSB%: Standard Deviation of Daily Cheyne Stokes Breathing Percentage; SWiM: Synthesis Without Meta-analysis.

### GRADE assessment by CPAP-CSR metric domain families

1. Presence Metrics (binary CSR detection)—Moderate certainty: Evidence from Prigent et al. (2022) indicates that incident CPAP-CSR detection is strongly associated with SCEs, with large effect sizes (unadjusted OR 13.66; adjusted OR 5.74; *p* < .001) at 12 months. Certainty was downgraded for risk of bias, as CSR-positive participants underwent prospective investigations while CSR-negative participants were assessed only retrospectively, introducing detection bias. The evidence was also downgraded for imprecision, reflecting modest sample sizes and single-centre design. No downgrades were applied for inconsistency or indirectness, as CSR presence directly aligns with the review’s HF-risk construct. Overall certainty: moderate.
2. Burden Metrics (CSR%, PB%, CSR-Positive Nights)—Moderate certainty: Across Prigent et al. (2025), Ullah et al. (2023), and Saito & Takamatsu (2022), CSR burden consistently correlated with HF severity and adverse outcomes. In Prigent et al. (2025), both CSR^+^ nights and CSR% were higher in SCE-positive patients, with CSR^+^ nights independently associated with SCE risk (OR 1.05; *p* = .017). Ullah et al. (2023) showed PB% was substantially higher in HFrEF vs HFnmEF and predicted HF hospitalisation while PAP adherence and HFrEF were associated with mortality. Certainty was downgraded for risk of bias, as these are observational designs with residual confounding, and for imprecision due to modest sample sizes. Despite this, consistency of direction across studies supported a moderate rating.
3. Morphology Metrics (Cycle Length, Breath Duration, Inter-Cycle Variability)—Moderate certainty: Midelet et al. (2023), demonstrated multiple morphological features distinguishing HF-CSR from non-HF periodic breathing with strong associations (e.g. cycle length OR 1.14 per second; 95% CI 1.07-1.21), with a low risk of bias. Saito and Takamatsu (2022)’s cycle-length findings were concordant, showing CL > 68.9 s discriminated HF from high-burden controls with AUC ≈ 0.95. Certainty was downgraded for risk of bias given reliance on observational data. Consistency and effect sizes avoided further downgrades. Certainty: moderate.
4. Short-Term Temporal Instability Metrics (SD-CSB%)—Low certainty: Temporal instability metrics, particularly SD-CSB%, demonstrated strong discrimination for imminent HF exacerbation in Saito & Takamatsu (2022) (AUC = 0.92). However, certainty was downgraded for serious risk of bias, as the evidence derives from a small, retrospective case–control study with marked selection bias, inadequate control of confounding, partially matched comparators, and unclear statistical methods for temporal comparisons. Additional downgrades were applied for indirectness, reflecting a restricted, high-CSB baseline population that does not represent the broader HF-OSA cohort, and for imprecision due to small sample size and event-limited inference. Overall certainty: low.
5. Long-Term Trajectory Metrics (CSR Progression Over Months/Years)—Moderate certainty: Prigent et al. (2025) demonstrated that CSR burden increased significantly only in CVD-CSR patients, while non-CVD CSR remained stable. All second-year SCEs occurred in the CVD-CSR subgroup, and CSR burden in the final 90-day window was associated with SCE occurrence. Certainty was downgraded for risk of bias (observational, non-randomized) and imprecision due to modest sample size and limited number of events. No downgrades for indirectness or inconsistency were made due to physiological coherence. Therefore: moderate.
6. Aetiology-Stratified CSR (CVD-CSR vs Non-CVD CSR)—Moderate certainty: Aetiology-stratified CSR behaves differently across studies. In Prigent et al. (2025), only CVD-CSR displayed progressive burden and all SCEs occurred in this subgroup. Midelet et al. (2023) supports this mechanistically, demonstrating structural waveform differences between HF-CSR and non-HF CSR. Downgrades were applied for indirectness, due to reliance on retrospective adjudication of CSR origin and absence of external validation, and for risk of bias given observational study designs. Nonetheless, strong consistency across physiological and clinical signals supports moderate certainty. Table 8 below summarizes this study’s GRADE assessment.

**Table 8:** Summary of GRADE certainty of evidence by CPAP-CSR metric domains [48, 56, 57, 66, 67]

SWiM metric family	Summary of Findings	GRADE Certainty
1. Presence (incident CSR detection)	CSR presence is consistently associated with increased risk of serious cardiac events, with large effect sizes but some detection bias in the primary study.	**Moderate**
2. Burden (%CSR, PB%, CSR-positive nights)	Higher CPAP-derived CSR burden is associated with worse HF outcomes and severity markers across studies, though measured in observational cohorts.	**Moderate**
3. Morphology (cycle length, breath duration, INTER-CYCLE VARIABILITY)	Distinctive morphological patterns reliably discriminate heart-failure-related CPAP-CSR from non-heat failure related CPAP-CSR, albeit from a small sample—supported by convergent physiological evidence.	**Moderate**
4a. Short-term temporal instability (SD-CSB%)	Short-term instability precedes HF decompensation, but evidence is from a single small, high RoB study.	**Low**
4b. Long-term trajectory (CSR progression)	CSR burden increases over months only in cardiovascular CPAP-CSR and tracks long-term risk of SCEs but derived from a single observational cohort.	**Moderate**
5. Aetiology-stratified CSR (CVD-CSR vs non-CVD CSR)	Stratified cardiovascular CPAP-CSR shows clear prognostic significance while non-cardiovascular CPAP-CSR does not, supported by both clinical and signal-morphology data.	**Moderate**

Boldface in Table 8 indicates the column headings, and final GRADE certainty rating assigned to each SWiM metric family.

Abbreviations: CSR: Cheyne–Stokes Respiration; CVD: Cardiovascular Disease; GRADE: Grading of Recommendations Assessment, Development and Evaluation; HF: Heart Failure; PB: Periodic Breathing; RoB: Risk of Bias; SCEs: Serious Cardiac Events; SD-CSB%: Standard Deviation of Daily Cheyne Stokes Breathing Percentage; SWiM: Synthesis Without Meta-analysis.

## Discussion

In prospective telemonitoring, incident CSR was associated with large increases in the risk of serious cardiac events, even after accounting for age and cardiovascular comorbidity. Similarly, burden measures such as CSR-positive nights, CSR%, CSB%, and PB% scaled with markers of haemodynamic compromise, including BNP, LVEF and eGFR, and were predictive of heart-failure hospitalisation or cardiovascular events during follow-up. These findings support the long-standing physiological model in which central instability of breathing in HF emerges when circulatory delay and heightened chemoreflex sensitivity reach a critical threshold. That these patterns can be detected through routine CPAP monitoring, without access to polysomnography or gas-exchange measurements, has meaningful implications for large-scale early-warning strategies and broader patient care.

Beyond presence and burden, several studies demonstrated that CPAP-CSR morphology carries discriminative information not captured by duration-based indices alone. The length of the oscillation, inter-cycle regularity, and breath timing all differed consistently between HF-CPAP-CSR and non-HF-CPAP-CSR. These morphological signatures, align closely with the canonical CSR produced by prolonged circulation time, and they contrast with the more irregular oscillations induced by leak, residual upper-airway obstruction or medication effects. Importantly, morphology-based discrimination was seen both in detailed cycle-by-cycle analyses and in studies relying on simplified metrics such as cycle length, suggesting that HF imposes a robust and recognisable structure on CPAP. This distinction is clinically essential i.e. without discriminating HF-CPAP-CSR from non-HF-CPAP-CSR, risk estimation will be diluted by episodes that do not reflect cardiovascular physiology.

Temporary, short-term instability in CSR burden, captured by increases in SD-CSB% over days, rose sharply in the lead up to HF events, achieving discriminative accuracy approaching 0.92. Cycle length lengthened during the same period, further reinforcing the notion that CPAP-CSR trends with dynamic destabilisation of ventilatory patterns, mirroring haemodynamic deterioration. Long-term data from telemonitoring cohorts added a complementary perspective: CSR burden increased substantially over two years only in patients whose CPAP-CSR was stratified as being cardiovascular in origin, and these individuals accounted for all subsequent serious cardiac events which were all hospitalisations. These different time scales, acute instability over days, and the more chronic escalation over years, altogether map well onto the natural history of heart-failure progression, in which early perturbations in circulatory feedback precede overt clinical deterioration. Together, they support the hypothesis that CPAP-CSR may not only be a marker of disease, or severity, but an evolving signal that reflects the state of cardiorespiratory coupling in real time.

Aetiology-stratified analyses were particularly informative, showing that the prognostic meaning of CPAP-CSR is contingent upon the physiological mechanism generating it. Only CPAP-CSR attributed to cardiovascular disease demonstrated progressive escalation and predicted adverse outcomes; CPAP-CSR caused by mask leak, residual obstruction or medication effects showed no meaningful temporal progression and carried no cardiac risk. This implies that CPAP-CSR is most clinically useful when contextualized and interpreted within cardiovascular physiology. It also suggests that future device algorithms may require basic aetiology-discrimination capabilities to prevent false positives and enhance interpretability.

In summary, this review provides consistent evidence across multiple cohorts that CPAP-CSR metrics detect, discriminate and track heart-failure physiology. Presence and burden identify individuals at greater cardiac risk; morphology differentiates HF-related from non-HF CPAP-CSR; temporal dynamics anticipate clinical deterioration; and aetiological stratification enhances specificity. Collectively, these findings suggest that CPAP-CSR may serve as a scalable physiological biomarker for heart-failure surveillance, offering early, actionable insights into circulatory-ventilatory instability—using data already captured in routine clinical practice. For example, the identification of long (> 69 s), uniform/less variable CSR cycles may aid in recognising occult heart failure in patients previously diagnosed only with obstructive sleep apnoea. Furthermore, measuring these variables prospectively and longitudinally in a home setting could enable clinicians to anticipate adverse outcomes such as acute decompensation before patient symptoms become clinically apparent. The ability to detect, discriminate and track these CPAP-CSR variables has the potential to facilitate earlier therapeutic escalation in at-risk patients—potentially reducing the burden associated with emergency hospitalisations.

### Study limitations

Despite these convergent findings, important limitations warrant consideration. All included studies were observational and relied on proprietary algorithms without open access to signal-processing logic. Sample sizes were modest, particularly for waveform-level analyse. The aetiological classifications used in the AlertApnée cohort, while clinically persuasive, were retrospective and not independently validated. Furthermore, several associations may be influenced by residual confounding, i.e. patients with high CSR burden were often older, had more comorbidities and were more adherent to CPAP therapy. These issues do not undermine the central patterns observed, but they do restrict the certainty with which the evidence can be considered.

## Conclusions

This systematic review shows that CPAP-CSR is likely a clinically meaningful respiratory signature that reflects underlying heart-failure physiology. Although the included studies were observational and varied in methodology, the consistency of direction and physiological coherence of the findings suggest that CPAP-CSR holds significant potential as a scalable, non-invasive, and passively collected digital biomarker that complements existing heart-failure monitoring strategies. With millions of CPAP devices already in routine daily use, there is considerable potential to leverage CPAP-CSR for continuous monitoring and surveillance, early detection of decompensation, improved risk stratification, and personalized follow-up in patients with, or at-risk of heart failure. However, given the current evidence is limited to a small number of heterogeneous observational trials, the results should not yet be interpreted as suggesting readiness for immediate clinical deployment. Prospective interventional trials are essential to establish the safety and efficacy of applying CPAP-CSR variables as standalone digital biomarkers in clinical decision-making.

## Future considerations

The findings of this review indicate that CPAP-CSR could meaningfully extend heart-failure surveillance from episodic, clinic-based encounters to long term, passive, home-based physiological monitoring. From a health-systems perspective, scalability applies equally to research infrastructure and clinical deployment. CPAP devices already generate continuous, structured airflow data at population scale, offering a uniquely low-cost, widely distributed platform which can be telemonitored. To harness this expansive reach, future work must focus on data standardisation, clinically interpretable metrics, and integration with electronic health-record systems. Realising this potential requires coordinated advances across both research and clinical practice. Several interdependent areas warrant focused attention.

A priority for future research is multicentre, device-agnostic validation across different device brands. Current evidence is limited by single-centre cohorts with device-specific algorithms that vary in window length, smoothing, amplitude thresholds and definitions of respiratory cycles, albeit while following the waveform component of AASM—CSR scoring guidelines. Validation studies should benchmark CPAP-CSR metrics against polysomnography, cardiac imaging, natriuretic peptides and decompensation markers to define diagnostic thresholds, temporal trajectories and actionable ranges.

A second area is the need for methodological harmonisation in distinguishing HF-specific CPAP-CSR from non-cardiac variants. The strong divergence in temporal behaviour and morphology across aetiologies observed in this review underscores the necessity of embedding aetiology discrimination into both research protocols and device algorithms.

Algorithmic innovation represents the most transformative frontier for both research and clinical deployment. Midelet et al (2023) machine-learning framework provides proof of concept that granular CSR morphology can be extracted from CPAP airflow signals and used to distinguish HF-related CSR from non-HF periodic breathing. Extending this approach to integrate short-term instability (e.g. SD-CSB%), long-term trajectories of CSR-positive nights, and multi-cycle morphological signatures offers a path towards refined, multi-dimensional stratification models. Combining such machine learning-driven aetiology classification with burden metrics such as CSR-positive nights may yield hybrid indices with superior predictive value for HF evolution before overt decompensation. Incorporating contextual information such as leak patterns, pressure settings, adherence behaviour and patient-level HF parameters could further enhance discriminative performance.

Clinical translation will require parallel work in implementation science. Prospective interventional trials are needed to determine whether integrating CPAP-CSR surveillance into HF management pathways can reduce hospitalisations, mitigate decompensation or refine risk stratification. Trials should test practical alert thresholds, escalation pathways and integration with existing monitoring systems, including natriuretic peptide testing, remote pulmonary artery pressure sensors, cardiac rehabilitation telemonitoring and patient-reported symptoms. At the clinical-workflow level, feasible and scalable strategies are required to manage automated notifications, triage alerts and determine when a change in CSR trajectory warrants clinical review. Clinical guidelines will eventually need to define CSR-based triggers for intervention—ranging from medication optimisation and volume assessment to expedited echocardiography or cardiology review.

In summary, advancing CPAP-CSR from an observational respiratory signature to a clinically actionable HF biomarker will require harmonized definitions, multicentre validation, refined machine-learning models capable of stratifying CSR by, aetiology and severity, and rigorous testing of clinical utility. With these developments, CPAP-derived respiratory phenotyping could meaningfully contribute to precision monitoring in heart failure, enabling earlier identification of instability and more responsive, personalized care.

## Supplementary Material

zpag042_Supplemental_Files

## Data Availability

Data sharing is not applicable to this article as no new data were created or analysed in this study. All data extracted and synthesized are included in this published article and its supplementary information files.

## Notes

PROSPERO Registration ID: [CRD420251171330]
