# Supplementary material for: Cheyne–Stokes respiration detected via CPAP devices as a digital biomarker for heart failure in obstructive sleep apnoea: systematic review
Source: Sleep Adv. 2026 Apr 6;7(2):zpag042. doi: 10.1093/sleepadvances/zpag042 (PMC13156490; doi:10.1093/sleepadvances/zpag042)
Supplement: zpag042_Supplemental_Files [file zpag042_supplemental_files.zip › CPAP-CSR-HF_Supplementary_Materials_amended_02.04.2026_zpag042.pdf]

Cheyne-Stokes Respiration Detected via CPAP Devices as a Digital Biomarker for Heart Failure in Obstructive Sleep Apnoea: Systematic Review

Authors:

Nashe Marshall Mutombe, Kanchana Ekanayake, Chin Moi Chow

Institution:

Faculty of Health and Medicine, University of Sydney, Camperdown, NSW, 2006, Australia.

Corresponding author: Nashe Marshall Mutombe, Faculty of Health and Medicine, Susan Wakil Health Building (D18), University of Sydney, Camperdown, NSW 2006, Australia. Email: nashe.marshall@sydney.edu.au

Kanchana Ekanayake. Email: Kanchana.ekanyake@sydney.edu.au

Chin Moi Chow. Email: chin-moi.chow@sydney.edu.au

Contents

Table S1: PRISMA 2020 Checklist for CPAP-CSR-HF Systematic Review 3
Text File S1: Search Strategy for CPAP-CSR-HF Systematic Review 6
Dataset S1: Data Extraction Sheets for CPAP-CSR-HF Systematic Review 11
Table S2: JBI Critical Appraisal Checklist for CPAP-CSR-HF Systematic Review 51

Table S1: PRISMA 2020 Checklist for CPAP-CSR-HF Systematic Review

Section and Topic	Item #	Checklist item	Location where item is reported (page)
**TITLE**			
Title	1	Identify the report as a systematic review.	1
**ABSTRACT**			
Abstract	2	See the PRISMA 2020 for Abstracts checklist.	2-3
**INTRODUCTION**			
Rationale	3	Describe the rationale for the review in the context of existing knowledge.	4-12
Objectives	4	Provide an explicit statement of the objective(s) or question(s) the review addresses.	12
**METHODS**			
Eligibility criteria	5	Specify the inclusion and exclusion criteria for the review and how studies were grouped for the syntheses.	14; 17-18
Information sources	6	Specify all databases, registers, websites, organisations, reference lists and other sources searched or consulted to identify studies. Specify the date when each source was last searched or consulted.	13
Search strategy	7	Present the full search strategies for all databases, registers and websites, including any filters and limits used.	13; Supplementary: Text file S1
Selection process	8	Specify the methods used to decide whether a study met the inclusion criteria of the review, including how many reviewers screened each record and each report retrieved, whether they worked independently, and if applicable, details of automation tools used in the process.	15
Data collection process	9	Specify the methods used to collect data from reports, including how many reviewers collected data from each report, whether they worked independently, any processes for obtaining or confirming data from study investigators, and if applicable, details of automation tools used in the process.	15
Data items	10a	List and define all outcomes for which data were sought. Specify whether all results that were compatible with each outcome domain in each study were sought (e.g. for all measures, time points, analyses), and if not, the methods used to decide which results to collect.	15
	10b	List and define all other variables for which data were sought (e.g. participant and intervention characteristics, funding sources). Describe any assumptions made about any missing or unclear information.	15; Supplementary; Dataset S1
Study risk of bias assessment	11	Specify the methods used to assess risk of bias in the included studies, including details of the tool(s) used, how many reviewers assessed each study and whether they worked independently, and if applicable, details of automation tools used in the process.	15
Effect measures	12	Specify for each outcome the effect measure(s) (e.g. risk ratio, mean difference) used in the synthesis or presentation of results.	Page 18; Page 43 (Table 3); Page 48 (Table 6)

Section and Topic	Item #	Checklist item	Location where item is reported (page)
Synthesis methods	13a	Describe the processes used to decide which studies were eligible for each synthesis (e.g. tabulating the study intervention characteristics and comparing against the planned groups for each synthesis (item #5)).	Pages 17-18 (Grouping); Page 18 (Criteria)
	13b	Describe any methods required to prepare the data for presentation or synthesis, such as handling of missing summary statistics, or data conversions.	16-17
	13c	Describe any methods used to tabulate or visually display results of individual studies and syntheses.	Page 17; Page 39 (Figures 2-5); Pages 41-51 (Tables 2-8)
	13d	Describe any methods used to synthesize results and provide a rationale for the choice(s). If meta-analysis was performed, describe the model(s), method(s) to identify the presence and extent of statistical heterogeneity, and software package(s) used.	Page 17 (Rationale); Page 18 (Approach)
	13e	Describe any methods used to explore possible causes of heterogeneity among study results (e.g. subgroup analysis, meta-regression).	Not applicable - no meta-analysis
	13f	Describe any sensitivity analyses conducted to assess robustness of the synthesized results.	Not applicable
Reporting bias assessment	14	Describe any methods used to assess risk of bias due to missing results in a synthesis (arising from reporting biases).	Not applicable
Certainty assessment	15	Describe any methods used to assess certainty (or confidence) in the body of evidence for an outcome.	13
**RESULTS**			
Study selection	16a	Describe the results of the search and selection process, from the number of records identified in the search to the number of studies included in the review, ideally using a flow diagram.	19; Figure 1
	16b	Cite studies that might appear to meet the inclusion criteria, but which were excluded, and explain why they were excluded.	19; Figure 1
Study characteristics	17	Cite each included study and present its characteristics.	19-20; Tables 2-3
Risk of bias in studies	18	Present assessments of risk of bias for each included study.	20-21; Figures 2-5; Supplementary Table S2
Results of individual studies	19	For all outcomes, present, for each study: (a) summary statistics for each group (where appropriate) and (b) an effect estimate and its precision (e.g. confidence/credible interval), ideally using structured tables or plots.	Pages 43-45 (Table 3); Page 48 (Table 6)
Results of syntheses	20a	For each synthesis, briefly summarise the characteristics and risk of bias among contributing studies.	Page 22; Pages 46-47 (Tables 4-5)
	20b	Present results of all statistical syntheses conducted. If meta-analysis was done, present for each the summary estimate and its precision (e.g. confidence/credible interval) and measures of statistical heterogeneity. If comparing groups, describe the direction of the effect.	22-26; Page 48 (Table 6); Page 49 (Table 7)
	20c	Present results of all investigations of possible causes of heterogeneity among study results.	Not applicable

Section and Topic	Item #	Checklist item	Location where item is reported (page)
	20d	Present results of all sensitivity analyses conducted to assess the robustness of the synthesized results.	Not applicable
Reporting biases	21	Present assessments of risk of bias due to missing results (arising from reporting biases) for each synthesis assessed.	Not applicable
Certainty of evidence	22	Present assessments of certainty (or confidence) in the body of evidence for each outcome assessed.	26-28; Page 51 (Table 8)
**DISCUSSION**			
Discussion	23a	Provide a general interpretation of the results in the context of other evidence.	28-30
	23b	Discuss any limitations of the evidence included in the review.	30-31
	23c	Discuss any limitations of the review processes used.	30-31
	23d	Discuss implications of the results for practice, policy, and future research.	31-33
**OTHER INFORMATION**			
Registration and protocol	24a	Provide registration information for the review, including register name and registration number, or state that the review was not registered.	13; 34
	24b	Indicate where the review protocol can be accessed, or state that a protocol was not prepared.	34
	24c	Describe and explain any amendments to information provided at registration or in the protocol.	Not applicable
Support	25	Describe sources of financial or non-financial support for the review, and the role of the funders or sponsors in the review.	34
Competing interests	26	Declare any competing interests of review authors.	34
Availability of data, code and other materials	27	Report which of the following are publicly available and where they can be found: template data collection forms; data extracted from included studies; data used for all analyses; analytic code; any other materials used in the review.	34; 16

From: Page MJ, McKenzie JE, Bossuyt PM, Boutron I, Hoffmann TC, Mulrow CD, et al. The PRISMA 2020 statement: an updated guideline for reporting systematic reviews. BMJ 2021;372:n71. doi: 10.1136/bmj.n71. This work is licensed under CC BY 4.0. To view a copy of this license, visit https://creativecommons.org/licenses/by/4.0/

Text File S1: Search Strategy for CPAP-CSR-HF Systematic Review

MEDLINE (OvidSP)

Coverage: 1946 to June 13, 2025

1. Sleep Apnea, Obstructive/

2. (sleep apnea* or sleep-disordered breathing).mp.

3. 1 or 2

4. Cheyne-Stokes Respiration/ or Continuous Positive Airway Pressure/ or Sleep Apnea, Central/ or exp Positive-Pressure Respiration/ or Monitoring, Physiologic/

5. (Cheyne-Stoke* or central sleep apnea* or central sleep apnoea* or periodic breath* or Continuous Positive Airway Pressure or CPAP monitor* or auto-CPAP or PAP device* or Positive airway pressure monitor* or CPAP data or PAP therap* or obstructive* pressure respirat* or physiology monitoring or device* based monitor* or remote monitor* or tele* monitor* or home monitor* or respiratory event detect* or respiratory signal analysis* or airflow signal* or flow signal*).mp.

6. 4 or 5

7. exp Heart Failure/ or exp Carotid Artery Diseases/ or exp Cardiovascular Diseases/

8. (heart failure* or carotid artery disease* or cardiovascular event* or myocardial infarction* or stroke* or sudden cardiac death* or heart disease* or cardiac event* or systolic heart failure* or cardiovascular disease* or cardiac mortalit* or cardiac risk* or left ventricular dysfunction* or Carotid Artery* or left ventricular ejection fraction* or Atrial Fibrillation*).mp.

9. 7 or 8

10. 3 and 6 and 9

Embase (OvidSP)

Coverage: 1947 to June 11, 2025

1. obstructive sleep apnea/

2. (sleep apnea* or sleep-disordered breathing).mp.

3. 1 or 2

4. Cheyne Stokes breathing/ or continuous positive airway pressure/ or positive pressure ventilation/ or physiologic monitoring/ or central sleep apnea syndrome/

5. (Cheyne-Stoke* or central sleep apnea* or central sleep apnoea* or periodic breath* or Continuous Positive Airway Pressure or CPAP monitor* or auto-CPAP or PAP device* or Positive airway pressure monitor* or CPAP data or PAP therap* or obstructive* pressure respirat* or physiology monitoring or device* based monitor* or remote monitor* or tele* monitor* or home monitor* or respiratory event detect* or respiratory signal analysis* or airflow signal* or flow signal*).mp.

6. 4 or 5

7. exp heart failure/ or carotid artery disease/ or cardiovascular disease/

8. (heart failure* or carotid artery disease* or cardiovascular event* or myocardial infarction* or stroke* or sudden cardiac death* or heart disease* or cardiac event* or systolic heart failure* or cardiovascular disease* or cardiac mortalit* or cardiac risk* or left ventricular dysfunction* or Carotid Artery* or left ventricular ejection fraction* or Atrial Fibrillation*).mp.

9. 7 or 8

10. 3 and 6 and 9

Scopus (Elsevier)

TITLE-ABS-KEY ("sleep apnea*" OR "sleep-disordered breathing")

AND

TITLE-ABS-KEY (cheyne-stoke* OR "central sleep apnea*" OR "central sleep apnoea*" OR "periodic breath*" OR "Continuous Positive Airway Pressure" OR "CPAP monitor*" OR auto-cpap OR "PAP device*" OR "Positive airway pressure monitor*" OR "CPAP data" OR "PAP therap*" OR "obstructive* pressure respirat*" OR "physiology monitoring" OR "device* based monitor*" OR "remote monitor*" OR "tele* monitor*" OR "home monitor*" OR "respiratory event detect*" OR "respiratory signal analysis" OR "airflow signal*" OR "flow signal*")

AND

TITLE-ABS-KEY ("heart failure*" OR "carotid artery disease*" OR "cardiovascular event*" OR "myocardial infarction*" OR stroke* OR "sudden cardiac death*" OR "heart disease*" OR "cardiac event*" OR "systolic heart failure*" OR "cardiovascular disease*" OR "cardiac mortalit*" OR "cardiac risk*" OR "left ventricular dysfunction*" OR "Carotid Artery*" OR "left ventricular ejection fraction*" OR "Atrial Fibrillation*")

Web of Science Core Collection (Clarivate)

#1 TS=("sleep apnea*" OR "sleep-disordered breathing")

#2 TS=(cheyne-stoke* OR "central sleep apnea*" OR "central sleep apnoea*" OR "periodic breath*" OR "Continuous Positive Airway Pressure" OR "CPAP monitor*" OR auto-cpap OR "PAP device*" OR "Positive airway pressure monitor*" OR "CPAP data" OR "PAP therap*" OR "obstructive* pressure respirat*" OR "physiology monitoring" OR "device* based monitor*" OR "remote monitor*" OR "tele* monitor*" OR "home monitor*" OR "respiratory event detect*" OR "respiratory signal analysis" OR "airflow signal*" OR "flow signal*")

#3 TS=("heart failure*" OR "carotid artery disease*" OR "cardiovascular event*" OR "myocardial infarction*" OR stroke* OR "sudden cardiac death*" OR "heart disease*" OR "cardiac event*" OR "systolic heart failure*" OR "cardiovascular disease*" OR "cardiac mortalit*" OR "cardiac risk*" OR "left ventricular dysfunction*" OR "Carotid Artery*" OR "left ventricular ejection fraction*" OR "Atrial Fibrillation*")

#4 #1 AND #2 AND #3

CINAHL (EBSCOhost)

S1 (MH "Sleep Apnea, Obstructive")

S2 ("sleep apnea*" OR "sleep-disordered breathing")

S3 S1 OR S2

S4 (MH "Respiration Disorders+")

S5 (MH "Continuous Positive Airway Pressure")

S6 (MH "Sleep Apnea, Central+")

S7 (MH "Positive-Pressure Respiration, Intrinsic")

S8 (MH "Monitoring, Physiologic+")

S9 (Cheyne-Stoke* OR "Respiration Disorder*" OR "central sleep apnea*" OR "central sleep apnoea*" OR "periodic breath*" OR "Continuous Positive Airway Pressure" OR "CPAP monitor*" OR auto-CPAP OR "PAP device*" OR "Positive airway pressure monitor*" OR "CPAP data" OR "PAP therap*" OR "obstructive* pressure respirat*" OR "physiology monitoring" OR "device* based monitor*" OR "remote monitor*" OR "tele* monitor*" OR "home monitor*" OR "respiratory event detect*" OR "respiratory signal analysis" OR "airflow signal*" OR "flow signal*")

S10 S4 OR S5 OR S6 OR S7 OR S8 OR S9

S11 (MH "Heart Failure+")

S12 (MH "Carotid Artery Diseases+")

S13 (MH "Cardiovascular Diseases+")

S14 ("heart failure*" OR "carotid artery disease*" OR "cardiovascular event*" OR "myocardial infarction*" OR stroke* OR "sudden cardiac death*" OR "heart disease*" OR "cardiac event*" OR "systolic heart failure*" OR "cardiovascular disease*" OR "cardiac mortalit*" OR "cardiac risk*" OR "left ventricular dysfunction*" OR "Carotid Artery*" OR "left ventricular ejection fraction*" OR "Atrial Fibrillation*")

S15 S11 OR S12 OR S13 OR S14

S16 S3 AND S10 AND S15

Dataset S1: Data Extraction Sheets for CPAP-CSR-HF Systematic Review

Study ID	Title
Saito 2022	Cheyne-Stokes Breathing as a Predictive Indicator of Heart Failure in Patients With Obstructive Sleep Apnea; A Retrospective Case Control Study Using Continuous Positive Airway Pressure Remote Monitoring Data
Saito 2022	Cheyne-Stokes Breathing as a Predictive Indicator of Heart Failure in Patients With Obstructive Sleep Apnea; A Retrospective Case Control Study Using Continuous Positive Airway Pressure Remote Monitoring Data
Prigent 2022	CPAP telemonitoring can track Cheyne-Stokes respiration and detect serious cardiac events: The AlertApnee Study
Prigent 2022	CPAP telemonitoring can track Cheyne-Stokes respiration and detect serious cardiac events: The AlertApnee Study
Midelet 2023	Features of Cheyne-Stokes respiration automatically extracted from CPAP airflow signal raw data: Identification of discriminating features to detect heart failure
Midelet 2023	Features of Cheyne-Stokes respiration automatically extracted from CPAP airflow signal raw data: Identification of discriminating features to detect heart failure
Ullah 2023	High nocturnal periodic breathing reported by PAP adherence data predicts decompensation of heart failure
Ullah 2023	High nocturnal periodic breathing reported by PAP adherence data predicts decompensation of heart failure
Prigent 2025	Incident Cheyne-Stokes respiration occurring in CPAP-treated patients and cardiovascular risk: a 2-years prospective follow-up (The Alertapnee study)
Prigent 2025	Incident Cheyne-Stokes respiration occurring in CPAP-treated patients and cardiovascular risk: a 2-years prospective follow-up (The Alertapnee study)

Study ID	From	From2	Status	Intervention	Group name
Saito 2022	Reviewer 1	Reviewer 2	Complete	Exposure	HF present (AHF and CHF)
Saito 2022	Reviewer 1	Reviewer 2	Complete	Comparator	HF absent (Control)
Prigent 2022	Reviewer 1	Reviewer 2	Complete	Exposure	Patients whose sudden increase in AHI was associated with CSR^+
Prigent 2022	Reviewer 1	Reviewer 2	Complete	Comparator	Patients whose sudden increase in AHI was not associated with CSR^-
Midelet 2023	Reviewer 1	Reviewer 2	Complete	Exposure	Patients with CSR and HF
Midelet 2023	Reviewer 1	Reviewer 2	Complete	Comparator	Patients with CSR without HF
Ullah 2023	Reviewer 1	Reviewer 2	Complete	Exposure	HFrEF: LVEF < 40%
Ullah 2023	Reviewer 1	Reviewer 2	Complete	Comparator	HFnmEF: LVEF ≥ 40%
Prigent 2025	Reviewer 1	Reviewer 2	Complete	Exposure	CSR associated with cardiovascular aetiology (heart failure, arrhythmia)

Study ID	From	From2	Status	Intervention	Group name
Prigent 2025	Reviewer 1	Reviewer 2	Complete	Comparator	CSR related to non-cardiovascular aetiologies, including residual obstructive events, mask leaks, medications, and other causes such as unknown/undetermined causes or renal insufficiency.

Identification

Study ID	Sponsorship source
Saito 2022	The study received no external funding. It was conducted as part of routine clinical practice at Saito Naika Kokyukika, Mie Sleep Clinic, Ise-shi, Japan.
Saito 2022	The study received no external funding. It was conducted as part of routine clinical practice at Saito Naika Kokyukika, Mie Sleep Clinic, Ise-shi, Japan.
Prigent 2022	Regional Health Agency of Brittany (ARS Bretagne), Polyclinique Saint-Laurent (Rennes), Air Liquide Healthcare / VitalAire France, Inserm U1300 (HP2 Laboratory, University Grenoble Alpes), French National Research Agency (ANR-15-IDEX-02, ANR-19-P3IA-0003), and the Fondation du Souffle / MIAI @ Grenoble Alpes.
Prigent 2022	Regional Health Agency of Brittany (ARS Bretagne), Polyclinique Saint-Laurent (Rennes), Air Liquide Healthcare / VitalAire France, Inserm U1300 (HP2 Laboratory, University Grenoble Alpes), French National Research Agency (ANR-15-IDEX-02, ANR-19-P3IA-0003), and the Fondation du Souffle / MIAI @ Grenoble Alpes.
Midelet 2023	This work was supported by the French National Research Agency (ANR) through the “Investissements d’avenir” program (grant ANR-15-IDEX-02), and by the Grenoble Alpes University Foundation (“e-health and integrated care and trajectories medicine” and MIAI Artificial Intelligence Chairs of Excellence; grant ANR-19-P3IA-0003).<br>The authors also acknowledge support from the Convention Industrielle de Formation par la Recherche (grant 2020/0007).
Midelet 2023	This work was supported by the French National Research Agency (ANR) through the “Investissements d’avenir” program (grant ANR-15-IDEX-02), and by the Grenoble Alpes University Foundation (“e-health and integrated care and trajectories medicine” and MIAI Artificial Intelligence Chairs of Excellence; grant ANR-19-P3IA-0003).<br>The authors also acknowledge support from the Convention Industrielle de Formation par la Recherche (grant 2020/0007).
Ullah 2023	Work for this study was performed at the G.V. (Sonny) Montgomery VA Medical Center, Jackson, Mississippi, USA.
Ullah 2023	Work for this study was performed at the G.V. (Sonny) Montgomery VA Medical Center, Jackson, Mississippi, USA.
Prigent 2025	The study was funded by Vitalaire, a company involved in home healthcare and oxygen/CPAP services.
Prigent 2025	The study was funded by Vitalaire, a company involved in home healthcare and oxygen/CPAP services.

Study ID	Country	Setting
Saito 2022	Japan.	Single-centre sleep clinic (Saito Naika Kokyukika, Mie Sleep Clinic, Ise-shi, Japan); retrospective case–control study using CPAP telemonitoring data analyses.
Saito 2022	Japan.	Single-centre sleep clinic (Saito Naika Kokyukika, Mie Sleep Clinic, Ise-shi, Japan); retrospective case–control study using CPAP telemonitoring data analyses.
Prigent 2022	France.	Monocentric prospective cohort of OSA adults on CPAP with daily telemonitoring; hospital clinic + homecare provider (VitalAire).

Study ID	Country	Setting
Prigent 2022	France.	Monocentric prospective cohort of OSA adults on CPAP with daily telemonitoring; hospital clinic + homecare provider (VitalAire).
Midelet 2023	France.	Conducted within CHU Grenoble Alpes, Service Universitaire de Pneumologie et Physiologie, in collaboration with Probayes (Montbonnot-Saint-Martin, France) and Groupe Médical de Pneumologie, Polyclinique Saint-Laurent (Rennes, France).
Midelet 2023	France.	Conducted within CHU Grenoble Alpes, Service Universitaire de Pneumologie et Physiologie, in collaboration with Probayes (Montbonnot-Saint-Martin, France) and Groupe Médical de Pneumologie, Polyclinique Saint-Laurent (Rennes, France).
Ullah 2023	United States of America.	Sleep Clinic, G.V. (Sonny) Montgomery Veterans Affairs Medical Center, Jackson, Mississippi, USA.
Ullah 2023	United States of America.	Sleep Clinic, G.V. (Sonny) Montgomery Veterans Affairs Medical Center, Jackson, Mississippi, USA.
Prigent 2025	France.	CPAP-telemonitored cohort from Polyclinique Saint-Laurent, Rennes.
Prigent 2025	France.	CPAP-telemonitored cohort from Polyclinique Saint-Laurent, Rennes.

Study ID	Comments
Saito 2022	The authors declared no commercial or financial conflicts of interest.
Saito 2022	The authors declared no commercial or financial conflicts of interest.
Prigent 2022	Conflicts: A. Prigent (consultant for ResMed; speaker fees from VitalAire); J. Texereau (employee of VitalAire); N. Coquerel (grants from Novartis); J.-L. Pépin (grants from Air Liquide Healthcare). Others declared no conflicts.
Prigent 2022	Conflicts: A. Prigent (consultant for ResMed; speaker fees from VitalAire); J. Texereau (employee of VitalAire); N. Coquerel (grants from Novartis); J.-L. Pépin (grants from Air Liquide Healthcare). Others declared no conflicts.
Midelet 2023	The authors declared that they have no known competing financial interests or personal relationships that could have influenced the work.<br>The study complied with the Declaration of Helsinki and was approved by the French ethics committee “Ouest VI” (CPP 1083-HPS2).<br>All patients provided written informed consent for research use of their de-identified data.
Midelet 2023	The authors declared that they have no known competing financial interests or personal relationships that could have influenced the work.<br>The study complied with the Declaration of Helsinki and was approved by the French ethics committee “Ouest VI” (CPP 1083-HPS2).<br>All patients provided written informed consent for research use of their de-identified data.
Ullah 2023	The authors report no conflicts of interest. All authors have seen and approved this manuscript.
Ullah 2023	The authors report no conflicts of interest. All authors have seen and approved this manuscript.

Study ID	Comments
Prigent 2025	Arnaud Prigent (AP): Consultant for ResMed; received personal fees from Elia Medical, Air Liquide Santé, and payments for presentations from ResMed Bastide, SOS Oxygène, GSK, and Isis Medical; also received non-financial support from Air Liquide Santé, Asten Santé, SOS O_2, and Elia Medical, all outside the submitted work.<br><br>Joëlle Texereau (JT): Employee of Air Liquide Healthcare.<br><br>Jean-Louis Pépin (JLP): Supported by grants from the French National Research Agency, the Grenoble Alpes University Foundation, and the MIAI AI Cluster; received lecture fees or travel grants from ResMed, Philips, Jazz Pharmaceuticals, Agiradom, Bastide, and Bioprojet.<br><br>Sébastien Bailly, Renaud Gervais, Anne-Laure Serandour, and Régis Luraine: Declared no conflicts of interest.
Prigent 2025	Arnaud Prigent (AP): Consultant for ResMed; received personal fees from Elia Medical, Air Liquide Santé, and payments for presentations from ResMed Bastide, SOS Oxygène, GSK, and Isis Medical; also received non-financial support from Air Liquide Santé, Asten Santé, SOS O_2, and Elia Medical, all outside the submitted work.<br><br>Joëlle Texereau (JT): Employee of Air Liquide Healthcare.<br><br>Jean-Louis Pépin (JLP): Supported by grants from the French National Research Agency, the Grenoble Alpes University Foundation, and the MIAI AI Cluster; received lecture fees or travel grants from ResMed, Philips, Jazz Pharmaceuticals, Agiradom, Bastide, and Bioprojet.<br><br>Sébastien Bailly, Renaud Gervais, Anne-Laure Serandour, and Régis Luraine: Declared no conflicts of interest.

Study ID	Author's name	Institution
Saito 2022	Kimimasa Saito, MD.	Saito Naika Kokyukika, Mie Sleep Clinic, Ise-shi, Japan.
Saito 2022	Kimimasa Saito, MD.	Saito Naika Kokyukika, Mie Sleep Clinic, Ise-shi, Japan.
Prigent 2022	Arnaud Prigent, MD.	Groupe Médical de Pneumologie / Centre du sommeil, Polyclinique Saint-Laurent, Rennes, France.
Prigent 2022	Arnaud Prigent, MD.	Groupe Médical de Pneumologie / Centre du sommeil, Polyclinique Saint-Laurent, Rennes, France.
Midelet 2023	Alphanie Midelet, PhD (candidate).	Univ. Grenoble Alpes, Inserm U1300, CHU Grenoble Alpes, Service Universitaire de Pneumologie Physiologie, Grenoble, France;<br>and Probayes, Montbonnot-Saint-Martin, France.
Midelet 2023	Alphanie Midelet, PhD (candidate).	Univ. Grenoble Alpes, Inserm U1300, CHU Grenoble Alpes, Service Universitaire de Pneumologie Physiologie, Grenoble, France;<br>and Probayes, Montbonnot-Saint-Martin, France.
Ullah 2023	Mohammad I. Ullah, MD, MPH.	Department of Medicine, University of Mississippi Medical Center, Jackson, Mississippi;
Ullah 2023	Mohammad I. Ullah, MD, MPH.	Department of Medicine, University of Mississippi Medical Center, Jackson, Mississippi;
Prigent 2025	Arnaud Prigent, MD.	Polyclinique Saint-Laurent, Rennes, France.

Study ID	Author's name	Institution
Prigent 2025	Arnaud Prigent, MD.	Polyclinique Saint-Laurent, Rennes, France.

Study ID	Email	Address
Saito 2022	k1saito@carrot.ocn.ne.jp	Saito Naika Kokyukika, Mie Sleep Clinic, Ise-shi, Mie Prefecture, Japan.<br>(No street address is provided in the publication, only city and prefecture — avoid fabricating or extending with unrelated details.)
Saito 2022	k1saito@carrot.ocn.ne.jp	Saito Naika Kokyukika, Mie Sleep Clinic, Ise-shi, Mie Prefecture, Japan.<br>(No street address is provided in the publication, only city and prefecture — avoid fabricating or extending with unrelated details.)
Prigent 2022	dr.arnaudprigent@gmail.com	Polyclinique Saint-Laurent, Rennes, France.
Prigent 2022	dr.arnaudprigent@gmail.com	Polyclinique Saint-Laurent, Rennes, France.
Midelet 2023	alphanie.midelet@gmail.com	53 Avenue Jean Kuntzmann, 38330 Montbonnot-Saint-Martin, France.
Midelet 2023	alphanie.midelet@gmail.com	53 Avenue Jean Kuntzmann, 38330 Montbonnot-Saint-Martin, France.
Ullah 2023	mullah@umc.edu	2500 N State St, University of Mississippi Medical Center, Jackson, MS; Tel: (601) 984-5660; Fax: (601) 984-5566.
Ullah 2023	mullah@umc.edu	2500 N State St, University of Mississippi Medical Center, Jackson, MS; Tel: (601) 984-5660; Fax: (601) 984-5566.
Prigent 2025	dr.arnaudprigent@gmail.com /<br>arnaud.prigent@chu-rennes.fr	Groupe Médical de Pneumologie, Polyclinique Saint Laurent, 2 ter rue de<br>Saint, Laurent, Rennes 35000, France.
Prigent 2025	dr.arnaudprigent@gmail.com /<br>arnaud.prigent@chu-rennes.fr	Groupe Médical de Pneumologie, Polyclinique Saint Laurent, 2 ter rue de<br>Saint, Laurent, Rennes 35000, France.

Methods

Study ID	Design	Group
Saito 2022	Case-control study	Parallel group
Saito 2022	Case-control study	Parallel group
Prigent 2022	Prospective cohort study	
Prigent 2022	Prospective cohort study	
Midelet 2023	Retrospective cross-sectional diagnostic analysis (using data from the AlertApnée cohort).	

Study ID	Design	Group
Midelet 2023	Retrospective cross-sectional diagnostic analysis (using data from the AlertApnée cohort).	
Ullah 2023	Retrospective Cross-Sectional	
Ullah 2023	Retrospective Cross-Sectional	
Prigent 2025	Prospective cohort study	
Prigent 2025	Prospective cohort study	

Population

Study ID	Inclusion criteria	Exclusion criteria
Saito 2022	Sample population inclusion: CPAP-treated OSA patients at Mie Sleep Clinic who developed HF between July 2014–May 2021 (AHF or CHF; HF diagnosed by cardiologists).<br><br>Control group inclusion: From a prior CPAP cohort, OSA patients using Philips DreamStation Auto ≥1 year, with diagnosis criteria AHI ≥ 20 (PSG) or AHI ≥ 40 (OCST), and 30-day average CSB% ≥ 1% during Sept 2020.	Control group exclusions: Age ≥ 90; poor CPAP adherence (<50% days or <4 h/night); new/worsening HF, new/relapse AF, or other cardiovascular disease within ±3 months of the reference date (Jun–Nov 2020).
Saito 2022	Sample population inclusion: CPAP-treated OSA patients at Mie Sleep Clinic who developed HF between July 2014–May 2021 (AHF or CHF; HF diagnosed by cardiologists).<br><br>Control group inclusion: From a prior CPAP cohort, OSA patients using Philips DreamStation Auto ≥1 year, with diagnosis criteria AHI ≥ 20 (PSG) or AHI ≥ 40 (OCST), and 30-day average CSB% ≥ 1% during Sept 2020.	Control group exclusions: Age ≥ 90; poor CPAP adherence (<50% days or <4 h/night); new/worsening HF, new/relapse AF, or other cardiovascular disease within ±3 months of the reference date (Jun–Nov 2020).
Prigent 2022	Adults on CPAP for OSA followed at the study centre; OSA diagnosed by polygraphy/PSG; CPAP initiated for AHI > 30 or AHI > 15 with cardiovascular comorbidity; all on the same device (ResMed AirSense 10) and same home-care provider.	Short-term poor prognosis with risk of death within 1 year.
Prigent 2022	Adults on CPAP for OSA followed at the study centre; OSA diagnosed by polygraphy/PSG; CPAP initiated for AHI > 30 or AHI > 15 with cardiovascular comorbidity; all on the same device (ResMed AirSense 10) and same home-care provider.	Short-term poor prognosis with risk of death within 1 year.
Midelet 2023	Adults with CPAP-confirmed Cheyne–Stokes respiration (CSR) detected via device airflow signal; CSR episodes clinically classified by expert physicians as due to heart failure, atrial fibrillation, or other cardiovascular causes; availability of high-quality raw airflow signal data for analysis.	Poor-quality or incomplete airflow data; unclear or unclassifiable CSR cause; missing relevant clinical data.

Study ID	Inclusion criteria	Exclusion criteria
Midelet 2023	Adults with CPAP-confirmed Cheyne–Stokes respiration (CSR) detected via device airflow signal; CSR episodes clinically classified by expert physicians as due to heart failure, atrial fibrillation, or other cardiovascular causes; availability of high-quality raw airflow signal data for analysis.	Poor-quality or incomplete airflow data; unclear or unclassifiable CSR cause; missing relevant clinical data.
Ullah 2023	Veterans with OSA + history of HF seen at the VA sleep clinic (2016–2018) who had required baseline data available: demographics, PAP adherence, PB% over prior 30 days, BNP (≤30 days), creatinine/eGFR, and LVEF (≤180 days); on CPAP/BPAP with modem data recorded.	Receiving narcotic pain medication or on dialysis.
Ullah 2023	Veterans with OSA + history of HF seen at the VA sleep clinic (2016–2018) who had required baseline data available: demographics, PAP adherence, PB% over prior 30 days, BNP (≤30 days), creatinine/eGFR, and LVEF (≤180 days); on CPAP/BPAP with modem data recorded.	Receiving narcotic pain medication or on dialysis.
Prigent 2025	Patients were selected from the original - 555-patient AlertApnée study cohort. Patients who had ≥1 CSR episode during year 1 and were still on CPAP, invited for year-2 follow-up (n = 66 included).<br><br>The inclusion criteria maintained - (adults on CPAP for OSA, AHI > 30 or > 15 with cardiovascular comorbidity, all on the same ResMed Airsense 10 device, same provider, with daily telemonitoring).	AlertApnée study patients without CSR in year 1 or no longer on CPAP were not entered into this ancillary follow-up.
Prigent 2025	Patients were selected from the original - 555-patient AlertApnée study cohort. Patients who had ≥1 CSR episode during year 1 and were still on CPAP, invited for year-2 follow-up (n = 66 included).<br><br>The inclusion criteria maintained - (adults on CPAP for OSA, AHI > 30 or > 15 with cardiovascular comorbidity, all on the same ResMed Airsense 10 device, same provider, with daily telemonitoring).	AlertApnée study patients without CSR in year 1 or no longer on CPAP were not entered into this ancillary follow-up.

Study ID	Number of withdrawals	Reason for withdrawals	Total sample size
Saito 2022	N/A Retrospective	N/A Retrospective	182 total (33 HF [11 AHF, 22 CHF], 149 controls).
Saito 2022	N/A Retrospective	N/A Retrospective	182 total (33 HF [11 AHF, 22 CHF], 149 controls).
Prigent 2022	Telemonitoring stopped before 12 months in 48 cases.	Discontinuation of CPAP (n = 20), early termination due to recurrent alerts with well-identified reasons (n = 25), and deaths (n = 3).	Complete data were available for 516 of the 555 included patients.
Prigent 2022	Telemonitoring stopped before 12 months in 48 cases.	Discontinuation of CPAP (n = 20), early termination due to recurrent alerts with well-identified reasons (n = 25), and deaths (n = 3).	Complete data were available for 516 of the 555 included patients.

Study ID	Number of withdrawals	Reason for withdrawals	Total sample size
Midelet 2023	N/A. Retrospective analysis.	N/A. Retrospective analysis.	Total patients: 23 (HF-CSR = 9; non-HF CSR = 14) Total CSR episodes analysed: 139 (HF-CSR = 78; non-HF CSR = 61)
Midelet 2023	N/A. Retrospective analysis.	N/A. Retrospective analysis.	Total patients: 23 (HF-CSR = 9; non-HF CSR = 14) Total CSR episodes analysed: 139 (HF-CSR = 78; non-HF CSR = 61)
Ullah 2023	Not applicable (retrospective chart review). Outcomes were ascertained from records within 180 days after the index visit.	Not applicable (retrospective chart review).	115 patients with OSA and HF. If a patient had multiple eligible visits, the earliest was used as the index visit.
Ullah 2023	Not applicable (retrospective chart review). Outcomes were ascertained from records within 180 days after the index visit.	Not applicable (retrospective chart review).	115 patients with OSA and HF. If a patient had multiple eligible visits, the earliest was used as the index visit.
Prigent 2025	2	lost to follow-up out of 66 (3%)	66 patients (64 completed 2-year follow-up)
Prigent 2025	2	lost to follow-up out of 66 (3%)	66 patients (64 completed 2-year follow-up)

Population Baseline Characteristics

Study ID	Mean age (years) ± SD	Mean age (years) ± SD (Overall)	Male (%)	Male (%) (Overall)
Saito 2022	76.2 ± 5.6	≈ 69	≈ 85 %	> 80 %
Saito 2022	66.9 ± 12.5	≈ 69	≈ 80 %	> 80 %
Prigent 2022	73.9 ± 9.3	63.7 ± 12.9	86%	74%
Prigent 2022	63.7 ± 12.9	63.7 ± 12.9	74%	74%
Midelet 2023	79.0 [79.0 – 83.0] years	79.0 [75.5 – 83.0] years	9 (100 %)	22 (96 %)
Midelet 2023	79.0 [75.0 – 82.0] years	79.0 [75.5 – 83.0] years	13 (92 %)	22 (96 %)
Ullah 2023	69 ± 11	69 ± 11	94%	94%
Ullah 2023	-	69 ± 11	94%	94%
Prigent 2025	73.2 ± 9.3	73.2 ± 9.3	86.40%	86%
Prigent 2025	72.8 ± 10.1	73.2 ± 9.3	85%	86%

Study ID	BMI (kg/m²)	BMI (kg/m²) (Overall)	OSA Severity (AHI, events/hr)	OSA Severity (AHI, events/hr) (Overall)
Saito 2022	26.3 ± 3.8	≈ 27	≥ 20 (PSG) or ≥ 40 (OCST)	≥ 20 (PSG) or ≥ 40 (OCST)
Saito 2022	27.9 ± 5.0	≈ 27	≥ 20 (PSG) or ≥ 40 (OCST)	≥ 20 (PSG) or ≥ 40 (OCST)
Prigent 2022	30.3 ± 4.2	31.7 ± 6.0	46.7 ± 14.1	≈ 45–47
Prigent 2022	31.7 ± 6.0	31.7 ± 6.0	44.5 ± 18.4	≈ 45–47
Midelet 2023	28.7 [24.1 – 31.2]	28.7 [26.5 – 30.6]	Adults with moderate-to-severe OSA on CPAP therapy (AHI > 30, or AHI > 15 with cardiovascular comorbidity).	Adults with moderate-to-severe OSA on CPAP therapy (AHI > 30, or AHI > 15 with cardiovascular comorbidity).
Midelet 2023	27.4 [26.5 – 30.5]	28.7 [26.5 – 30.6]	Adults with moderate-to-severe OSA on CPAP therapy (AHI > 30, or AHI > 15 with cardiovascular comorbidity).	Adults with moderate-to-severe OSA on CPAP therapy (AHI > 30, or AHI > 15 with cardiovascular comorbidity).
Ullah 2023	31.2 ± 6.3	31.2 ± 6.3	38.7 ± 12.1	38.7 ± 12.1
Ullah 2023	-	31.2 ± 6.3	-	38.7 ± 12.1
Prigent 2025	30.3 ± 4.1	30.3 ± 4.1	46.6 ± 14.5	≈ 46
Prigent 2025	31.0 ± 5.0	30.3 ± 4.1	-	≈ 46

Study ID	Comorbidities	Comorbidities (Overall)
Saito 2022	HF (AHF or CHF), AF, CAD diagnosed by cardiologists	OSA patients on CPAP ≥ 1 year
Saito 2022	OSA without CV disease within ±3 months	OSA patients on CPAP ≥ 1 year
Prigent 2022	Higher CV burden (HF, AF, CAD more frequent)	57 % with CV disease overall
Prigent 2022	Lower CV risk profile	57 % with CV disease overall
Midelet 2023	Heart failure (n = 9); median LVEF 50 % [40–60]; NT-proBNP 2407 [1241–7589] pg/mL; Left atrial volume index 71 [62.5–81] mL/m²	HF and non-HF comorbidities.
Midelet 2023	Non-HF causes (n = 14): persistent obstructive apnoea (10), mask leaks (2), medication (1), renal insufficiency (1)	HF and non-HF comorbidities.
Ullah 2023	All HF patients; hypertension, CAD, AF common	All HF patients; hypertension, CAD, AF common
Ullah 2023	-	All HF patients; hypertension, CAD, AF common
Prigent 2025	Cardiovascular disease (including heart failure or arrhythmia) in 32 % of patients; non-cardiac CSR causes included residual OSA, mask leak, medication effects, or renal dysfunction.	Mixed CV and non-CV causes of CSR
Prigent 2025	Non-cardiac leaks, drug effects, renal issues	Mixed CV and non-CV causes of CSR

Study ID	Follow-up Duration	Follow-up Duration (Overall)
Saito 2022	CPAP monitoring over 30 days before HF onset; retrospective data available for up to 7 years (2014–2021)	CPAP monitoring over 30 days before HF onset (cases) or during September 2020 (controls); retrospective data spanning October 2007 – May 2021.
Saito 2022	CPAP monitoring over 30 days (September 2020) using telemonitoring data; clinical and echocardiographic data obtained May 2019 – November 2020.	CPAP monitoring over 30 days before HF onset (cases) or during September 2020 (controls); retrospective data spanning October 2007 – May 2021.
Prigent 2022	1 year	1 year
Prigent 2022	1 year	1 year
Midelet 2023	Up to 61 days between CSR data and cardiologic consultation (cross-sectional analysis only)	Up to 61 days between CSR data and cardiologic consultation (cross-sectional analysis only)
Midelet 2023	Up to 61 days between CSR data and cardiologic consultation (cross-sectional analysis only)	Up to 61 days between CSR data and cardiologic consultation (cross-sectional analysis only)
Ullah 2023	180 days	180 days
Ullah 2023	180 days	180 days
Prigent 2025	2 years	2 years
Prigent 2025	2 years	2 years

Study ID	Primary Outcome	Primary Outcome (Overall)
Saito 2022	Prediction of HF onset from CSB% and cycle length	Prediction of HF onset from CSB% and cycle length
Saito 2022	Prediction of HF onset from CSB% and cycle length	Prediction of HF onset from CSB% and cycle length
Prigent 2022	Serious cardiac events (arrhythmia, acute HF)	Serious cardiac events (arrhythmia, acute HF)
Prigent 2022	Serious cardiac events (arrhythmia, acute HF)	Serious cardiac events (arrhythmia, acute HF)
Midelet 2023	The feasibility of automatically extracted Cheyne–Stokes respiration (CSR) airflow signal features from CPAP raw data to discriminate between CSR associated with heart failure (HF) and CSR due to non-HF causes.	The feasibility of automatically extracted Cheyne–Stokes respiration (CSR) airflow signal features from CPAP raw data to discriminate between CSR associated with heart failure (HF) and CSR due to non-HF causes.
Midelet 2023	The feasibility of automatically extracted Cheyne–Stokes respiration (CSR) airflow signal features from CPAP raw data to discriminate between CSR associated with heart failure (HF) and CSR due to non-HF causes.	The feasibility of automatically extracted Cheyne–Stokes respiration (CSR) airflow signal features from CPAP raw data to discriminate between CSR associated with heart failure (HF) and CSR due to non-HF causes.
Ullah 2023	HF decompensation requiring hospitalisation	HF decompensation requiring hospitalisation
Ullah 2023	HF decompensation requiring hospitalisation	HF decompensation requiring hospitalisation

Study ID	Primary Outcome	Primary Outcome (Overall)
Prigent 2025	Association between cardiovascular morbidity–related CSR burden (CSR frequency and %) and significant cardiac events (SCEs).	Association between cardiovascular morbidity–related CSR burden (CSR frequency and %) and significant cardiac events (SCEs).
Prigent 2025	Association between cardiovascular morbidity–related CSR burden (CSR frequency and %) and significant cardiac events (SCEs).	Association between cardiovascular morbidity–related CSR burden (CSR frequency and %) and significant cardiac events (SCEs).

Study ID	Withdrawals / Attrition	Withdrawals / Attrition (Overall)
Saito 2022	Not reported	N/A
Saito 2022	Not reported	N/A
Prigent 2022	20 discontinued CPAP, 25 recurrent alerts, 3 deaths	48 total (8.6 %)
Prigent 2022	-	48 total (8.6 %)
Midelet 2023	None reported (23 patients retained in final analysis)	N/A. Retrospective analysis.
Midelet 2023	N/A. Retrospective analysis.	N/A. Retrospective analysis.
Ullah 2023	N/A (retrospective dataset)	-
Ullah 2023	-	-
Prigent 2025	2 lost to follow-up (3 %)	64 / 66 completed
Prigent 2025	-	64 / 66 completed

Study ID	CPAP device details	CPAP device details (Overall)
Saito 2022	Philips DreamStation Auto	Philips DreamStation Auto
Saito 2022	Philips DreamStation Auto	Philips DreamStation Auto
Prigent 2022	ResMed AirSense 10 AutoSet (S10)	ResMed AirSense 10 AutoSet (S10)
Prigent 2022	ResMed AirSense 10 AutoSet (S10)	ResMed AirSense 10 AutoSet (S10)
Midelet 2023	CPAP devices with SD-card raw airflow data (rlk binary format parsed to .csv) Data analysed retrospectively using the rlk-parser feature-extraction code (Python).	ResMed telemonitored CPAP units (through AlertApnée study)
Midelet 2023	CPAP devices with SD-card raw airflow data (rlk binary format parsed to .csv). Data analysed retrospectively using the rlk-parser feature-extraction code (Python).	ResMed telemonitored CPAP units (through AlertApnée study)
Ullah 2023	Philips Respironics DreamStation with modem	Philips Respironics DreamStation with modem

Study ID	CPAP device details	CPAP device details (Overall)
Ullah 2023	Philips Respironics DreamStation with modem	Philips Respironics DreamStation with modem
Prigent 2025	ResMed AirSense 10	ResMed AirSense 10
Prigent 2025	ResMed AirSense 10	ResMed AirSense 10

Study ID	Mode of data acquisition	Mode of data acquisition (Overall)
Saito 2022	EncoreAnywhere remote telemonitoring	Cloud-based data collection
Saito 2022	EncoreAnywhere remote telemonitoring	Cloud-based data collection
Prigent 2022	Daily telemonitoring (via home-care provider)	Daily telemonitoring (via home-care provider)
Prigent 2022	Daily telemonitoring (via home-care provider)	Daily telemonitoring (via home-care provider)
Midelet 2023	Raw airflow signal from device memory card (downloaded after rAHI alert > 7 events/h for ≥ 2 nights or ≥ 15 events/h for 1 night)	Automated data download and Python-based signal processing
Midelet 2023	Raw airflow signal from device memory card (downloaded after rAHI alert > 7 events/h for ≥ 2 nights or ≥ 15 events/h for 1 night)	Automated data download and Python-based signal processing
Ullah 2023	Automated telemetry upload (VA database)	Electronic records + modem logs
Ullah 2023	-	Electronic records + modem logs
Prigent 2025	Telemonitoring database (ResMed AirView)	Telemonitoring database (ResMed AirView)
Prigent 2025	Telemonitoring database (ResMed AirView)	Telemonitoring database (ResMed AirView)

Study ID	CPAP therapy adherence	CPAP therapy adherence (Overall)
Saito 2022	≥ 4 h/night on > 50 % of nights (inclusion threshold)	Good adherence across cohort
Saito 2022	≥ 4 h/night on > 50 % of nights (inclusion threshold)	Good adherence across cohort
Prigent 2022	Mean 5.9 ± 1.4 h/night	≈ 6 h/night overall
Prigent 2022	Similar	≈ 6 h/night overall
Midelet 2023	The AlertApnée cohort included only adherent patients (≥ 4 h/night on ≥ 70 % of nights), and ongoing CPAP use at alert was required.	The AlertApnée cohort included only adherent patients (≥ 4 h/night on ≥ 70 % of nights), and ongoing CPAP use at alert was required.
Midelet 2023	The AlertApnée cohort included only adherent patients (≥ 4 h/night on ≥ 70 % of nights), and ongoing CPAP use at alert was required.	The AlertApnée cohort included only adherent patients (≥ 4 h/night on ≥ 70 % of nights), and ongoing CPAP use at alert was required.

Study ID	CPAP therapy adherence	CPAP therapy adherence (Overall)
Ullah 2023	Median 86 % of nights > 4 h	86 % > 4 h nights (average ~5.9 h/night)
Ullah 2023	-	86 % > 4 h nights (average ~5.9 h/night)
Prigent 2025	Stable throughout 2 years (~6 h/night)	Stable use maintained
Prigent 2025	Stable throughout 2 years (~6 h/night)	Stable use maintained

Study ID	LVEF (%)	LVEF (%) (Overall)	Heart Failure (HF)	Heart Failure (HF) (Overall)
Saito 2022	Categorised (HFrEF < 40 %, HFmrEF 40–49 %, HFpEF ≥ 50 %) – mean LVEF not reported.	LVEF categorised only for heart-failure patients; not reported for controls.	8.4 ± 9.2	22 (66.7)
Saito 2022	LVEF not applicable – no heart failure diagnosed.	LVEF categorised only for heart-failure patients; not reported for controls.	0	22 (66.7)
Prigent 2022	-	-	12 (16.2%)	27 (4.9%)
Prigent 2022	-	-	15 (3.1%)	27 (4.9%)
Midelet 2023	50.0 [40.0, 60.0]	60.0 [42.0, 66.0]	9	23
Midelet 2023	68.0 [61.0, 68.0]	60.0 [42.0, 66.0]	14	23
Ullah 2023	24.63 ± 8.44 %	41 ± 14 (overall estimate)	-	115 total (41 HFrEF, 74 HFnmEF)
Ullah 2023	52.59 ± 5.37 %	41 ± 14 (overall estimate)	-	115 total (41 HFrEF, 74 HFnmEF)
Prigent 2025	-	-	-	-
Prigent 2025	-	-	-	-

Intervention Characteristics

Study ID	CSR/PB characteristics	HF characteristics
Saito 2022	Elevated SD of CSB % and/or prolonged cycle length (CL)	
Saito 2022	Elevated SD of CSB % and/or prolonged cycle length (CL)	
Prigent 2022	Incident CSR within 12 months	
Prigent 2022	Incident CSR within 12 months	

Study ID	CSR/PB characteristics	HF characteristics
Midelet 2023	CSR features detected (Cycle length ↑; CSR duration ↑; amplitude variation ↑; breath duration ↓; inter-cycle variability ↓; expiratory/inspiratory ratio ↑.	Presence of clinically diagnosed heart failure
Midelet 2023	CSR features detected (Cycle length ↑; CSR duration ↑; amplitude variation ↑; breath duration ↓; inter-cycle variability ↓; expiratory/inspiratory ratio ↑.	Absence of clinically diagnosed heart failure
Ullah 2023		
Ullah 2023		
Prigent 2025	Frequency of nights with cardiovascular morbidity - related CSR	
Prigent 2025	Frequency of nights with cardiovascular morbidity - related CSR	

Reported Outcomes and Result Notes

Study ID	Reported outcomes in study
Saito 2022	Probability of Cycle length (CL) during stable period - Discriminating CHF vs Controls, Probability that SD CSR% predicts HF onset (HF vs Control)
Saito 2022	Probability of Cycle length (CL) during stable period - Discriminating CHF vs Controls, Probability that SD CSR% predicts HF onset (HF vs Control)
Prigent 2022	Primary Outcomes D (SCEs (acute HF or arrhythmia) requiring therapeutic intervention.)
Prigent 2022	Primary Outcomes D (SCEs (acute HF or arrhythmia) requiring therapeutic intervention.)
Midelet 2023	Diagnostic Accuracy of Automated CSR Feature analysis for Heart Failure
Midelet 2023	Diagnostic Accuracy of Automated CSR Feature analysis for Heart Failure
Ullah 2023	Primary Outcomes D (Hospitalisations due to acute HF decompensation occurring within 180 days after that index visit.)
Ullah 2023	Primary Outcomes D (Hospitalisations due to acute HF decompensation occurring within 180 days after that index visit.)
Prigent 2025	Primary Outcomes D (Occurrence of an SCE (acute HF or arrhythmia) requiring therapeutic intervention)
Prigent 2025	Primary Outcomes D (Occurrence of an SCE (acute HF or arrhythmia) requiring therapeutic intervention)

Study ID	Result data notes
Saito 2022	Primary outcome analysis:<br>Multivariate logistic regression including SD of CSB % and CL as independent variables.<br>AUC = 0.919 (for SD CSB%) and AUC = 0.954 (for CL).<br><br>ROC for SD CSB% predicting HF onset: AUC = 0.919 (95% CI 0.874–0.964) p < 0.001. cut-off = 3.275% (Sensitivity 0.879; Specificity 0.886).<br>ROC for CL predicting HF onset: AUC = 0.954 (95% CI 0.910–0.997) p < 0.001. Cut-off = 68.9 s (Sensitivity 0.864; Specificity 0.909).<br><br>Additional analyses found CSB % increased progressively in the days leading up to heart-failure onset, and CL was significantly longer during CHF exacerbation than during stable periods (p = 0.004). No multivariable, combined, or predefined subgroup analyses were performed.<br><br>NB: Data in data tables below is representative only due to Covidence reporting limitations. Use Data in Notes.
Saito 2022	Primary outcome analysis:<br>Multivariate logistic regression including SD of CSB % and CL as independent variables.<br>AUC = 0.919 (for SD CSB%) and AUC = 0.954 (for CL).<br><br>ROC for SD CSB% predicting HF onset: AUC = 0.919 (95% CI 0.874–0.964) p < 0.001. cut-off = 3.275% (Sensitivity 0.879; Specificity 0.886).<br>ROC for CL predicting HF onset: AUC = 0.954 (95% CI 0.910–0.997) p < 0.001. Cut-off = 68.9 s (Sensitivity 0.864; Specificity 0.909).<br><br>Additional analyses found CSB % increased progressively in the days leading up to heart-failure onset, and CL was significantly longer during CHF exacerbation than during stable periods (p = 0.004). No multivariable, combined, or predefined subgroup analyses were performed.<br><br>NB: Data in data tables below is representative only due to Covidence reporting limitations. Use Data in Notes.
Prigent 2022	Primary outcome analysis:<br>A multivariable logistic regression model was adjusted for age, dyslipidaemia, and the presence of ≥ 1 cardiovascular comorbidity (heart failure, valvular cardiopathy, coronary artery disease, or atrial fibrillation).<br>Detection of Cheyne–Stokes respiration (CSR) by CPAP telemonitoring was independently associated with a higher risk of serious cardiac events:<br>Adjusted OR = 5.74 (95% CI 2.08 – 16.83), p < 0.001.<br><br>In univariable logistic regression, the presence of CSR was strongly associated with incident SCEs:<br>Unadjusted OR = 13.7 (95% CI 5.1 – 38.9), p < 0.001.<br><br>Subgroup analysis:<br>he paper also notes that, within the CSR^{+} subgroup, CSR-related variables (e.g., number of CSR nights and percentage of CSR time) correlated significantly with the occurrence of SCEs (p = 0.012 and p = 0.008, respectively). no OR reported for CSR^{+} and CSR-.

Study ID	Result data notes
Prigent 2022	Primary outcome analysis:<br>A multivariable logistic regression model was adjusted for age, dyslipidaemia, and the presence of ≥ 1 cardiovascular comorbidity (heart failure, valvular cardiopathy, coronary artery disease, or atrial fibrillation).<br>Detection of Cheyne–Stokes respiration (CSR) by CPAP telemonitoring was independently associated with a higher risk of serious cardiac events:<br>Adjusted OR = 5.74 (95% CI 2.08 – 16.83), $p < 0.001$.<br><br>In univariable logistic regression, the presence of CSR was strongly associated with incident SCEs:<br>Unadjusted OR = 13.7 (95% CI 5.1 – 38.9), $p < 0.001$.<br><br>Subgroup analysis:<br>he paper also notes that, within the CSR^{+} subgroup, CSR-related variables (e.g., number of CSR nights and percentage of CSR time) correlated significantly with the occurrence of SCEs ($p = 0.012$ and $p = 0.008$, respectively). no OR reported for CSR^{+} and CSR-.
Midelet 2023	Primary outcome analysis:<br>Multivariable binary logistic-regression, adjusted for within-patient clustering (Huber-White robust errors).<br>Only statistically significant predictive features were applied in the final model i.e.,<br><br>Cycle length ↑ → HF-CSR more likely.<br><br>Amplitude variation ↑ → HF-CSR more likely.<br><br>Average breath duration ↓ → HF-CSR more likely.<br><br>Inter-cycle variability ↓ → HF-CSR more likely.<br><br>Model performance was evaluated by repeated cross-validation, yielding a mean classification accuracy of 0.72 ± 0.13, recall (sensitivity) 0.78 ± 0.22, and F1-score 0.72 ± 0.19.<br><br>Predicted probability > 0.5 → classified as positive.<br><br>longer, smoother, more regular CSR cycles (with strong waxing–waning but stable pattern) signal heart-failure-related CSR.

Study ID	Result data notes
Midelet 2023	Primary outcome analysis:<br>Multivariable binary logistic-regression, adjusted for within-patient clustering (Huber-White robust errors).<br>Only statistically significant predictive features were applied in the final model i.e.,<br><br>Cycle length ↑ → HF-CSR more likely.<br><br>Amplitude variation ↑ → HF-CSR more likely.<br><br>Average breath duration ↓ → HF-CSR more likely.<br><br>Inter-cycle variability ↓ → HF-CSR more likely.<br><br>Model performance was evaluated by repeated cross-validation, yielding a mean classification accuracy of 0.72 ± 0.13, recall (sensitivity) 0.78 ± 0.22, and F1-score 0.72 ± 0.19.<br><br>Predicted probability > 0.5 → classified as positive.<br><br>longer, smoother, more regular CSR cycles (with strong waxing–waning but stable pattern) signal heart-failure-related CSR.
Ullah 2023	Primary outcome analysis:<br>Negative binomial regression predicting number of hospitalisations from acute heart failure within 180 days of the index visit/ earliest routine follow-up appointment in the sleep clinic between January 1, 2016 and November 8, 2018.<br>Univariate model: PB (%) - IRR 1.02 (95 % CI 1.01 – 1.03), p = 0.001.<br><br>Bivariate model: After adjusting one covariate at a time (PB kept constant).<br>Race (Black vs White): IRR 1.89, p = 0.033<br>Every +10 % PAP adherence: IRR 0.78, p < 0.001<br>HFrEF vs HFnmEF: IRR 7.45, p < 0.001<br><br>Secondary outcome (mortality within 180 days):<br>Binary logistic regression models were run with PB % as the primary predictor. PB % was retained and each covariate (race, PAP adherence, HF type, age, atrial fibrillation) was added one at a time to test for confounding effects..<br>Periodic breathing (%): OR 1.02 per 1 % increase; p = 0.089 (95 % CI not reported).<br>PAP adherence (per 10 % increase): OR 0.86; p = 0.043.<br>HFrEF vs HFnmEF: OR 2.78; p = 0.045.<br><br>NB: Race, age and AF were not statistically significant.<br>NB: There is no aggregate multivariable model combining all covariates - each was entered separately while PB% remained fixed.

Study ID	Result data notes
Ullah 2023	Primary outcome analysis:<br>Negative binomial regression predicting number of hospitalisations from acute heart failure within 180 days of the index visit/ earliest routine follow-up appointment in the sleep clinic between January 1, 2016 and November 8, 2018.<br>Univariate model: PB (%) - IRR 1.02 (95 % CI 1.01 – 1.03), p = 0.001.<br><br>Bivariate model: After adjusting one covariate at a time (PB kept constant).<br>Race (Black vs White): IRR 1.89, p = 0.033<br>Every +10 % PAP adherence: IRR 0.78, p < 0.001<br>HFrEF vs HFnmEF: IRR 7.45, p < 0.001<br><br>Secondary outcome (mortality within 180 days):<br>Binary logistic regression models were run with PB % as the primary predictor. PB % was retained and each covariate (race, PAP adherence, HF type, age, atrial fibrillation) was added one at a time to test for confounding effects..<br>Periodic breathing (%): OR 1.02 per 1 % increase; p = 0.089 (95 % CI not reported).<br>PAP adherence (per 10 % increase): OR 0.86; p = 0.043.<br>HFrEF vs HFnmEF: OR 2.78; p = 0.045.<br><br>NB: Race, age and AF were not statistically significant.<br>NB: There is no aggregate multivariable model combining all covariates - each was entered separately while PB% remained fixed.
Prigent 2025	Primary outcome analysis:<br><br>Univariate logistic regression -<br>OR = 1.05 (95 % CI 1.01–1.09, p = 0.017) per CSR night<br><br>The number of nights with cardiovascular morbidity–related CSR was independently associated with an increased risk of SCE<br><br>CSR nights per 90 days:<br>Mann–Whitney U test<br>Median 48 in SCE (IQR = 35) versus 9.5 (IQR = 27.8) No SCE (p = 0.012).<br><br>Mean CSR% per 90 days:<br>Mann–Whitney U test<br>Median CSR 13.8 % (IQR = 13.7) in SCE versus 6.1 % (IQR = 4.5) No SCE (p = 0.008).<br><br>Both CSR nights' frequency and CSR% were significantly associated with SCE. however the 2 metrics were significantly collinear (Spearman r = 0.53, p < 0.001).

Study ID	Result data notes
Prigent 2025	Primary outcome analysis:<br><br>Univariate logistic regression -<br>OR = 1.05 (95 % CI 1.01–1.09, p = 0.017) per CSR night<br><br>The number of nights with cardiovascular morbidity–related CSR was independently associated with an increased risk of SCE<br><br>CSR nights per 90 days:<br>Mann–Whitney U test<br>Median 48 in SCE (IQR = 35) versus 9.5 (IQR = 27.8) No SCE (p = 0.012).<br><br>Mean CSR% per 90 days:<br>Mann–Whitney U test<br>Median CSR 13.8 % (IQR = 13.7) in SCE versus 6.1 % (IQR = 4.5) No SCE (p = 0.008).<br><br>Both CSR nights' frequency and CSR% were significantly associated with SCE. however the 2 metrics were significantly collinear (Spearman r = 0.53, p < 0.001).

Primary Outcomes D (Hospitalisations due to acute HF decompensation occurring within 180 days after that index visit.)

Study ID	Reported name	Outcome type
Saito 2022		
Saito 2022		
Prigent 2022		
Prigent 2022		
Midelet 2023		
Midelet 2023		
Ullah 2023	Hospitalisations due to acute HF decompensation occurring within 180 days after that index visit.	Dichotomous
Ullah 2023	Hospitalisations due to acute HF decompensation occurring within 180 days after that index visit.	Dichotomous
Prigent 2025		

Study ID	Reported name	Outcome type
Prigent 2025		

Study ID	Reported against	Reported as	Reference arm	Outcome group
Saito 2022	Standard		Not applicable	Primary
Saito 2022	Standard		Not applicable	Primary
Prigent 2022	Standard		Not applicable	Primary
Prigent 2022	Standard		Not applicable	Primary
Midelet 2023	Standard		Not applicable	Primary
Midelet 2023	Standard		Not applicable	Primary
Ullah 2023	Standard	RR, 95% CI, p-value	Not applicable	Primary
Ullah 2023	Standard	RR, 95% CI, p-value	Not applicable	Primary
Prigent 2025	Standard		Not applicable	Primary
Prigent 2025	Standard		Not applicable	Primary

Study ID	Data value	Timepoints	(End of study) Reported time
Saito 2022		End of study	
Saito 2022		End of study	
Prigent 2022		End of study	12 months
Prigent 2022		End of study	12 months
Midelet 2023		End of study	
Midelet 2023		End of study	

Study ID	Data value	Timepoints	(End of study) Reported time
Ullah 2023	Endpoint	End of study	180 days
Ullah 2023	Endpoint	End of study	180 days
Prigent 2025		End of study	24 months
Prigent 2025		End of study	24 months

Study ID	RR	Lower	Upper	p-value
Saito 2022				
Saito 2022				
Prigent 2022				
Prigent 2022				
Midelet 2023				
Midelet 2023				
Ullah 2023	1.02	1.01	1.03	0.001
Ullah 2023				
Prigent 2025				
Prigent 2025				

Primary Outcomes D (Occurrence of an SCE (acute HF or arrhythmia) requiring therapeutic intervention)

Study ID	Reported name	Outcome type
Saito 2022		
Saito 2022		

Study ID	Reported name	Outcome type
Prigent 2022		
Prigent 2022		
Midelet 2023		
Midelet 2023		
Ullah 2023		
Ullah 2023		
Prigent 2025	Occurrence of an SCE (acute HF or arrhythmia) requiring therapeutic intervention	Dichotomous
Prigent 2025	Occurrence of an SCE (acute HF or arrhythmia) requiring therapeutic intervention	Dichotomous

Study ID	Reported against	Reported as	Reference arm	Outcome group
Saito 2022	Standard		Not applicable	Primary
Saito 2022	Standard		Not applicable	Primary
Prigent 2022	Standard		Not applicable	Primary
Prigent 2022	Standard		Not applicable	Primary
Midelet 2023	Standard		Not applicable	Primary
Midelet 2023	Standard		Not applicable	Primary
Ullah 2023	Standard		Not applicable	Primary
Ullah 2023	Standard		Not applicable	Primary
Prigent 2025	Standard	OR, 95% CI, p-value	Not applicable	Primary
Prigent 2025	Standard	OR, 95% CI, p-value	Not applicable	Primary

Study ID	Data value	Timepoints	(End of study) Reported time
Saito 2022		End of study	
Saito 2022		End of study	
Prigent 2022		End of study	12 months
Prigent 2022		End of study	12 months
Midelet 2023		End of study	
Midelet 2023		End of study	
Ullah 2023		End of study	180 days
Ullah 2023		End of study	180 days
Prigent 2025	Endpoint	End of study	24 months
Prigent 2025	Endpoint	End of study	24 months

Study ID	OR	Lower	Upper	p-value
Saito 2022				
Saito 2022				
Prigent 2022				
Prigent 2022				
Midelet 2023				
Midelet 2023				
Ullah 2023				
Ullah 2023				
Prigent 2025	1.05	1.01	1.09	0.017

Study ID	OR	Lower	Upper	p-value
Prigent 2025				

Primary Outcomes D (SCEs (acute HF or arrhythmia) requiring therapeutic intervention.)

Study ID	Reported name	Outcome type
Saito 2022		
Saito 2022		
Prigent 2022	SCEs (acute HF or arrhythmia) requiring therapeutic intervention.	Dichotomous
Prigent 2022	SCEs (acute HF or arrhythmia) requiring therapeutic intervention.	Dichotomous
Midelet 2023		
Midelet 2023		
Ullah 2023		
Ullah 2023		
Prigent 2025		
Prigent 2025		

Study ID	Reported against	Reported as	Reference arm	Outcome group
Saito 2022	Standard		Not applicable	Primary
Saito 2022	Standard		Not applicable	Primary
Prigent 2022	Standard	OR, 95% CI, p-value	Not applicable	Primary
Prigent 2022	Standard	OR, 95% CI, p-value	Not applicable	Primary
Midelet 2023	Standard		Not applicable	Primary
Midelet 2023	Standard		Not applicable	Primary

Study ID	Reported against	Reported as	Reference arm	Outcome group
Ullah 2023	Standard		Not applicable	Primary
Ullah 2023	Standard		Not applicable	Primary
Prigent 2025	Standard		Not applicable	Primary
Prigent 2025	Standard		Not applicable	Primary

Study ID	Data value	Timepoints	(End of study) Reported time
Saito 2022		End of study	
Saito 2022		End of study	
Prigent 2022	Endpoint	End of study	12 months
Prigent 2022	Endpoint	End of study	12 months
Midelet 2023		End of study	
Midelet 2023		End of study	
Ullah 2023		End of study	180 days
Ullah 2023		End of study	180 days
Prigent 2025		End of study	24 months
Prigent 2025		End of study	24 months

Study ID	OR	Lower	Upper	p-value
Saito 2022				
Saito 2022				
Prigent 2022	5.7	2.08	16.83	0.001

Study ID	OR	Lower	Upper	p-value
Prigent 2022				
Midelet 2023				
Midelet 2023				
Ullah 2023				
Ullah 2023				
Prigent 2025				
Prigent 2025				

Probability of Cycle length (CL) during stable period - Discriminating CHF vs Controls

Study ID	Outcome type
Saito 2022	Continuous
Saito 2022	Continuous
Prigent 2022	
Prigent 2022	
Midelet 2023	
Midelet 2023	
Ullah 2023	
Ullah 2023	
Prigent 2025	
Prigent 2025	

Study ID	Reported against	Reported as	Reference arm	Outcome group
Saito 2022	Standard	95% CI, p-value, Total	Not applicable	Primary
Saito 2022	Standard	95% CI, p-value, Total	Not applicable	Primary
Prigent 2022	Standard		Not applicable	Primary
Prigent 2022	Standard		Not applicable	Primary
Midelet 2023	Standard		Not applicable	Primary
Midelet 2023	Standard		Not applicable	Primary
Ullah 2023	Standard		Not applicable	Primary
Ullah 2023	Standard		Not applicable	Primary
Prigent 2025	Standard		Not applicable	Primary
Prigent 2025	Standard		Not applicable	Primary

Study ID	Data value	Timepoints
Saito 2022	Endpoint	End of study
Saito 2022	Endpoint	End of study
Prigent 2022		End of study
Prigent 2022		End of study
Midelet 2023		End of study
Midelet 2023		End of study
Ullah 2023		End of study
Ullah 2023		End of study
Prigent 2025		End of study

Study ID	Data value	Timepoints
Prigent 2025		End of study

Study ID	Lower	Upper	Total	p-value
Saito 2022	0.91	0.997	954	0.001
Saito 2022				
Prigent 2022				
Prigent 2022				
Midelet 2023				
Midelet 2023				
Ullah 2023				
Ullah 2023				
Prigent 2025				
Prigent 2025				

Diagnostic Accuracy of Automated CSR Feature analysis for Heart Failure

Study ID	Reported name	Outcome type
Saito 2022		
Saito 2022		
Prigent 2022		
Prigent 2022		
Midelet 2023	Probability of CSR features predicting HF (aggregate classification performance).	Continuous

Study ID	Reported name	Outcome type
Midelet 2023	Probability of CSR features predicting HF (aggregate classification performance).	Continuous
Ullah 2023		
Ullah 2023		
Prigent 2025		
Prigent 2025		

Study ID	Reported against	Reported as	Reference arm	Outcome group
Saito 2022	Standard		Not applicable	Primary
Saito 2022	Standard		Not applicable	Primary
Prigent 2022	Standard		Not applicable	Primary
Prigent 2022	Standard		Not applicable	Primary
Midelet 2023	Standard	mean, SD, p-value	Not applicable	Primary
Midelet 2023	Standard	mean, SD, p-value	Not applicable	Primary
Ullah 2023	Standard		Not applicable	Primary
Ullah 2023	Standard		Not applicable	Primary
Prigent 2025	Standard		Not applicable	Primary
Prigent 2025	Standard		Not applicable	Primary

Study ID	Direction	Data value	Timepoints
Saito 2022			End of study
Saito 2022			End of study

Study ID	Direction	Data value	Timepoints
Prigent 2022			End of study
Prigent 2022			End of study
Midelet 2023	Higher is better	Endpoint	End of study
Midelet 2023	Higher is better	Endpoint	End of study
Ullah 2023			End of study
Ullah 2023			End of study
Prigent 2025			End of study
Prigent 2025			End of study

Study ID	mean	SD	p-value
Saito 2022			
Saito 2022			
Prigent 2022			
Prigent 2022			
Midelet 2023	0.72	0.13	0.05
Midelet 2023	0.72	0.13	0.05
Ullah 2023			
Ullah 2023			
Prigent 2025			
Prigent 2025			

Probability that SD CSR% predicts HF onset (HF vs Control)

Study ID	Outcome type
Saito 2022	Continuous
Saito 2022	Continuous
Prigent 2022	
Prigent 2022	
Midelet 2023	
Midelet 2023	
Ullah 2023	
Ullah 2023	
Prigent 2025	
Prigent 2025	

Study ID	Reported against	Reported as	Reference arm	Outcome group
Saito 2022	Standard	95% CI, p-value, Total	Not applicable	Primary
Saito 2022	Standard	95% CI, p-value, Total	Not applicable	Primary
Prigent 2022	Standard		Not applicable	Primary
Prigent 2022	Standard		Not applicable	Primary
Midelet 2023	Standard		Not applicable	Primary
Midelet 2023	Standard		Not applicable	Primary
Ullah 2023	Standard		Not applicable	Primary
Ullah 2023	Standard		Not applicable	Primary

Study ID	Reported against	Reported as	Reference arm	Outcome group
Prigent 2025	Standard		Not applicable	Primary
Prigent 2025	Standard		Not applicable	Primary

Study ID	Data value	Timepoints
Saito 2022	Endpoint	End of study
Saito 2022	Endpoint	End of study
Prigent 2022		End of study
Prigent 2022		End of study
Midelet 2023		End of study
Midelet 2023		End of study
Ullah 2023		End of study
Ullah 2023		End of study
Prigent 2025		End of study
Prigent 2025		End of study

Study ID	Lower	Upper	Total	p-value
Saito 2022	0.874	0.964	919	0.001
Saito 2022				
Prigent 2022				
Prigent 2022				
Midelet 2023				

Study ID	Lower	Upper	Total	p-value
Midelet 2023				
Ullah 2023				
Ullah 2023				
Prigent 2025				
Prigent 2025				

Study-level data extraction for Midelet et al. (2023)

Category	Variable/Feature	Measure	Value	95% CI / IQR	p-value	Notes
Multivariate Model	Cycle length	OR	1.14	1.07 – 1.21	<0.05	Significant. Per 1-sec increase
Multivariate Model	Duration of CSR episode	OR	1.04	0.98 – 1.11	NS	Per 1-min increase
Multivariate Model	Breaths avg duration	OR	0.57	0.35 – 0.91	<0.05	Significant. Per 1-sec increase
Multivariate Model	Inter-cycle variability	OR	0.41	0.20 – 0.84	<0.05	Significant. Per 1-unit increase
Multivariate Model	Expiratory-inspiratory amplitude ratio	OR	2.51	0.30 – 20.83	NS	CI crosses 1
Multivariate Model	Big breath amplitude ratio	OR	0.76	0.35 – 1.67	NS	CI crosses 1
Multivariate Model	Modulation amplitude	OR	0.01	0.00 – 0.24	<0.05	Significant. Value appears contradictory to text description ("greater variation... associated with HF"); text may refer to raw feature distribution vs adjusted model
Model Performance	Accuracy	Mean	0.72	± 0.13	-	
Model Performance	Recall	Mean	0.78	± 0.22	-	
Model Performance	F1-score	Mean	0.72	± 0.19	-	
Univariate (Table 3)	Cycle length (s)	Median (HF)	64.8	56.3 – 74.8	<0.01	Higher in HF (vs 50.6 in Non-HF)
Univariate (Table 3)	Breath count	Median (HF)	22	18.0 – 26.0	<0.01	Higher in HF (vs 15.0 in Non-HF)
Univariate (Table 3)	Expiratory-inspiratory amplitude ratio	Median (HF)	1.3	1.1 – 1.4	<0.01	Higher in HF (vs 1.1 in Non-HF)
Univariate (Table 3)	Big breath amplitude ratio	Median (HF)	2.6	2.3 – 3.3	0.01	Lower in HF (vs 3.2 in Non-HF)

Category	Variable/Feature	Measure	Value	95% CI / IQR	p-value	Notes
Univariate (Table 3)	Duration of CSR episode (s)	Median (HF)	1468.5	1023 – 1862	0.28	No significant difference (vs 1351.2)
Univariate (Table 3)	Breath, avg. duration (s)	Median (HF)	2.9	1.7 – 4.1	0.28	No significant difference (vs 3.9)
Univariate (Table 3)	Inter-cycle variability (DTW)	Median (HF)	3.2	2.7 – 3.9	0.31	No significant difference (vs 3.1)
Univariate (Table 3)	Amplitude variation index	Median (HF)	0.8	0.6 – 0.9	0.85	No significant difference (vs 0.8)

Study-level data extraction for Saito et al. (2022)

Study	Outcome	AUC	Lower 95% CI	Upper 95% CI	p-value	p-symbol	Cut-off	Sensitivity	Specificity	Notes	Effect type
Saito & Takamatsu, 2022	SD CSB% predicting HF onset	0.919	0.874	0.964	0.001	<	3.275	0.879	0.886	p<0.001; Fig 5B	
Saito & Takamatsu, 2022	CL discriminating CHF vs Controls	0.954	0.91	0.997	0.001	<	68.9	0.864	0.909	p<0.001; Fig 6; seconds	
Saito & Takamatsu, 2022	CSB% temporal variation				0.001	<				Group and Group×Time p<0.001; Fig 4	Two-way RM ANOVA
Saito & Takamatsu, 2022	CL stable vs exacerbation				0.004					79.3±11.9 vs 85.1±11.5 s	Wilcoxon paired

Study-level data extraction for Ullah et al. (2023)

Section	Predictor	Effect	Value	Lower 95% CI	Upper 95% CI	p-value	p-symbol	Notes
Primary – Hospitalizations (NegBin)	PB% (per 1%)	IRR	1.02			0.001		Univariate.
Primary – Hospitalizations (NegBin, bivariate)	Race (Black vs White)	IRR	1.89			0.033		PB% retained; one covariate at a time.
Primary – Hospitalizations (NegBin, bivariate)	HF type (HFrEF vs HFnmEF)	IRR	7.45			0.001	<	Reported p<0.001.
Primary – Hospitalizations (NegBin, bivariate)	PAP adherence (per 10%)	IRR	0.78			0.001	<	Protective.
Secondary – Mortality (Logistic)	PB% (per 1%)	OR	1.02			0.089		NS.
Secondary – Mortality (Logistic, bivariate)	PAP adherence (per 10%)	OR	0.86			0.043		Protective.
Secondary – Mortality (Logistic, bivariate)	HF type (HFrEF vs HFnmEF)	OR	2.78			0.045		Significant.
Additional – Correlations	PB% vs BNP	r	0.447			0.01	<	
Additional – Correlations	PB% vs Hospitalizations	r	0.331			0.01	<	
Additional – Correlations	PB% vs LVEF	r	-0.423			0.01	<	
Additional – Correlations	PB% vs eGFR	r	-0.246			0.01	<	

Section	Predictor	Effect	Value	Lower 95% CI	Upper 95% CI	p-value	p-symbol	Notes
Additional – Linear model (PB% outcome)	LVEF (β)	β	-0.514			0.001	<	
Additional – Linear model (PB% outcome)	BNP (β)	β	0.012			0.001	<	
Additional – Linear model (PB% outcome)	eGFR (β)	β	-0.115			0.035		

Study-level data extraction for Saito et al. (2022)

Section	Outcome	Effect	Value	Lower 95% CI	Upper 95% CI	p-value	p-symbol	Notes
Primary – SCE at 12 months	Incident CSR (vs none)	OR	13.7	5.1	38.9	0.01	<	Univariable logistic regression.
Primary – SCE at 12 months	Incident CSR (adjusted)	OR	5.74	2.08	16.83	0.001	<	Adjusted for age, dyslipidaemia, ≥1 CV comorbidity.

Study-level data extraction for Prigent et al. (2025)

Section	Outcome	Effect	Value	Lower 95% CI	Upper 95% CI	p-value	p-symbol	Notes
Y1→Y2 change (overall)	Number of CSR-positive nights (Within-patient change in) (Year 2 vs Year 1) (paired 90 day windows)	Wilcoxon				0.025		Median 8.5 (Y2) vs 6 (Y1).
Y1→Y2 change (CV etiology)	Number of CSR-positive nights (Cardiovascular Aetiology Subgroup = SCE) (Y1 vs Y2)	Wilcoxon				0.006		Median 37 (Y2) vs 19 (Y1).
Year-2 SCE vs no-SCE	Number of CSR-positive nights (SCE vs no SCE)	Mann-Whitney U				0.012		Median 48 (SCE) vs 9.5. (non-SCE)
Year-2 SCE vs no-SCE	Mean percentage of sleep time spent in CSR over final 90-day window (SCE vs no SCE)(on CSR + nights)	Mann-Whitney U				0.008		Median 13.8% vs 6.1%.
Logistic model (Y2 SCE)	Odds of serious cardiac event per additional CSR-positive night (univariable)	OR	1.05	1.01	1.09	0.017		Univariable logistic regression.

Table S2: JBI Critical Appraisal Checklist for CPAP-CSR-HF Systematic Review

Scoring key

mark note	Yes:1 = low risk	No:2 = high risk	Unclear:3 = Unclear risk	Not applicable:4 = Not applicable

JBI CRITICAL APPRAISAL CHECKLIST FOR COHORT STUDIES

Study / Record	1. Were the two groups similar and recruited from the same population?	2. Were the exposures measured similarly to assign people to both exposed and unexposed groups?	3. Was the exposure measured in a valid and reliable way?	4. Were confounding factors identified?	5. Were strategies to deal with confounding factors stated?	6. Were the groups/participants free of the outcome at the start of the study (or at the moment of exposure)?	7. Were the outcomes measured in a valid and reliable way?	8. Was the follow up time reported and sufficient to be long enough for outcomes to occur?	9. Was follow up complete, and if not, were the reasons to loss to follow up described and explored?	10. Were strategies to address incomplete follow up utilized?	11. Was appropriate statistical analysis used?	Overall appraisal (Include / Exclude / Seek info)	Comments	Comments 2
Prigent et al., 2022	1	1	1	1	1	1	3	1	1	1	1	Include		• The CSR- group was assessed passively and retrospectively, versus the CSR+ whose CSR and potential SCEs were assessed for following an increased AHI alert - altogether potentially causing some selection bias.
Prigent et al., 2025	1	1	1	1	1	1	1	1	1	1	1	Include		

JBI CRITICAL APPRAISAL CHECKLIST FOR CASE CONTROL STUDIES

Study / Record	1. Were the groups comparable other than the presence of disease in cases or the absence of disease in controls?	2. Were cases and controls matched appropriately?	3. Were the same criteria used for identification of cases and controls?	4. Was exposure measured in a standard, valid and reliable way?	5. Was exposure measured in the same way for cases and controls?	6. Were confounding factors identified?	7. Were strategies to deal with confounding factors stated?	8. Were outcomes assessed in a standard, valid and reliable way for cases and controls?	9. Was the exposure period of interest long enough to be meaningful?	10. Was appropriate statistical analysis used?	Overall appraisal (Include / Exclude / Seek info)	Comments	Comments 2
Saito & Takamatsu, 2022	2	3 (partial)	1	1	1	2	2	1	1	2	Meets inclusion criteria, but classified as high risk of bias. (Interpret findings cautiously).	Strengths<br>• They used ROC curves to identify cut-off points for CSB% and cycle length (CL), which is appropriate for diagnostic performance evaluation.<br>• Matching was attempted for CSB%, BMI, and sex in a subset (CHF vs. controls), which is a step toward reducing confounding.<br>Issues<br>1. Confounding Variables Not Fully Addressed<br>o The initial comparison between HF and non-HF groups seems to have differences in baseline characteristics (e.g., age, comorbidities, severity of OSA). These were not	>1CSR% represent (at-risk) controls with CSR burden.

												adjusted for in the main analysis.<br>o Matching was only partial (BMI, sex, CSB%), and only for CHF vs. controls—not for AHF or the entire HF group.<br>2. Statistical Methods Not Clearly Reported<br>o The description does not mention multivariable regression (e.g., logistic regression), which is standard for case-control studies to adjust for confounders.<br>o Chronologic changes of CSB% were compared among groups, but the method (ANOVA? repeated measures?) is unclear.<br>3. Selection Bias<br>o Controls were selected based on CSB% ≥ 1%, which may introduce bias because this criterion is related to the exposure of interest.	

JBI CRITICAL APPRAISAL CHECKLIST FOR ANALYTICAL CROSS SECTIONAL STUDIES

Study / Record	1. Were the criteria for inclusion in the sample clearly defined?	2. Were the study subjects and the setting described in detail?	3. Was the exposure measured in a valid and reliable way?	4. Were objective, standard criteria used for measurement of the condition?	5. Were confounding factors identified?	6. Were strategies to deal with confounding factors stated?	7. Were the outcomes measured in a valid and reliable way?	8. Was appropriate statistical analysis used?	Overall appraisal (Include / Exclude / Seek info)	Comments	Comments 2
Midelet et al., 2023	1	1	1	1	1	1	1	1	Include	Design: Prospective observational study (sometimes loosely called a cohort because of follow-up). Analysis: Cross-sectional (descriptive of CSR episodes rather than comparing exposures over time).	
Ullah et al., 2023	1	1	1	1	1	1	1	1	Include	This is a retrospective cross-sectional study because it analyses data from a single point in time (the present review of medical records) but uses data that was collected at different points in the past (e.g., labs from the previous 30 days). While the data covers different time points, the study itself does not follow a cohort over time but instead captures a snapshot of the patients at the moment the data was compiled from their medical records.	All the CPAP-CSR are following the same AASM criteria for scoring CSB -part (a) CSR classification.
