# Supplementary material for: Cheyne–Stokes respiration detected via CPAP devices as a digital biomarker for heart failure in obstructive sleep apnoea: systematic review
Source: Sleep Adv. 2026 Apr 6;7(2):zpag042. doi: 10.1093/sleepadvances/zpag042 (PMC13156490; doi:10.1093/sleepadvances/zpag042)
Supplement: zpag042_Supplemental_Files [file zpag042_supplemental_files.zip › Supplementary_Materials_-_List_of_Captions_zpag042.docx]

# Supplementary Materials: List of Captions

Text File S1: Search Strategy for CPAP-CSR-HF systematic review.

Dataset S1: Data Extraction Sheets for CPAP-CSR-HF systematic review.

Table S1: PRISMA 2020 Checklist for CPAP-CSR-HF systematic review.

Table S2: JBI Critical Appraisal Checklist for CPAP-CSR-HF systematic review.
